# Targeting USP10/SCD1 axis by RAF265 suppresses lipogenesis and induced ferroptosis in head and neck squamous cell carcinoma

**DOI:** 10.1038/s41420-026-03180-1

**Published:** 2026-06-02

**Authors:** Shuhan Shi, Xinyan Sun, Xintong Kui, Zixin Gao, Jiajun Song, Chuanchun han, Xiaoyan Zhang, Dapeng Liang, Xiaojie Li, Yuan Wang

**Affiliations:** 1 The Second Affiliated Hospital, Dalian Medical University, Dalian, PR China; 2 School of Stomatology, Dalian Medical University, Dalian, PR China; 3 The First Affiliated Hospital, Dalian Medical University, Dalian, PR China; 4 Institute of Cancer Stem Cells, Dalian Medical University, Dalian, PR China; 5 Qilu Medical University, Zibo, PR China

**Keywords:** Head and neck cancer, Cell death

## Abstract

Ubiquitin-specific peptidase 10 (USP10) has been implicated in the development of various cancers, including head and neck squamous cell carcinoma (HNSCC). Nevertheless, the precise mechanisms through which USP10 functions in HNSCC are not fully understood. In this work, we demonstrate that USP10 acts as a deubiquitinating enzyme for stearoyl-CoA desaturase 1 (SCD1), directly interacting with SCD1 to remove ubiquitin chains, thereby enhancing its protein stability. This stabilization results in elevated SCD1 levels and then promotes lipogenesis of monounsaturated fatty acids and reduced ferroptotic cell death in HNSCC cells. Furthermore, our data reveal that the transcription factor E2F4 activates USP10 expression by binding to its promoter region in HNSCC. Notably, we discover that RAF265, a compound already approved by the FDA, effectively inhibits USP10 activity, leading to decreased SCD1 expression and lipogenesis, which then suppresses tumor growth and enhances ferroptosis in both in vitro and in vivo models of HNSCC. Collectively, these results underscore the critical role of the E2F4/USP10/SCD1 pathway in modulating ferroptosis and driving HNSCC progression, suggesting that targeting this axis with RAF265 may offer a promising strategy for therapeutic intervention in this malignancy.

## Introduction

HNSCC represents the most prevalent type of head and neck cancer, primarily originating from the larynx, pharynx, and oral cavity [1, 2]. Although significant progress has been made in treatment approaches, including targeted drugs, surgical interventions, and immunotherapies, the overall survival outcomes for HNSCC patients remain largely unchanged [3]. Thus, there is an urgent need to explore the molecular underpinnings of this disease and identify novel therapeutic avenues.

A key regulatory mechanism in cellular protein dynamics is ubiquitination, a reversible posttranslational modification mediated by the ubiquitin (Ub) system that governs the stability, localization, and activity of numerous proteins [4, 5]. The removal of ubiquitin moieties is catalyzed by deubiquitinating enzymes (DUBs), which play crucial roles in maintaining protein homeostasis [6, 7]. To date, around 100 human DUBs have been identified and classified into six major families: USP, JAMM, MJD, OTU, MINDY, and UCH [8, 9].

Among these, USP10 belongs to the ubiquitin-specific protease (USP) family and has emerged as a critical player in cancer development [10, 11]. Accumulating evidence indicates its dual role depending on the cellular context. For instance, Shi et al. demonstrated that USP10 stabilizes IGF2BP1, thereby facilitating metastasis in breast cancer [12], while Yang et al. showed that USP10 enhances TCF4 stability to drive tumor progression in the same malignancy [13]. In hepatocellular carcinoma, Zhu et al. found that USP10 activates the YAP/TAZ signaling axis through stabilization, promoting oncogenesis [14]. Conversely, in esophageal squamous cell carcinoma (ESCC), suppression of USP10 leads to reduced ANLN levels and attenuated tumorigenicity [15]. Additionally, USP10 contributes to ESCC aggressiveness and resistance to radiation by preserving HDAC7 and PARP protein stability [16]. Notably, Yuan et al. reported that USP10 can also exert tumor-suppressive effects by increasing the stability of p53 and PTEN [17–20]. Our previous work further revealed that USP10 interacts with KLF4, enhancing its accumulation and inhibiting lung cancer progression [21]. More recently, Zhang et al. showed that USP10 upregulates POLR2A expression and suppresses ferroptosis in HNSCC. Shi et al. found that USP10 stabilizes BAZ1A to promote disease progression.

Despite growing recognition of USP10’s involvement in HNSCC, the precise mechanisms through which it regulates tumor biology are not yet fully understood. In this study, we identify SCD1 as a novel interacting partner of USP10. We demonstrate that USP10 enhances SCD1 protein stability, consequently inhibiting ferroptotic cell death and driving the malignant phenotype of HNSCC. Moreover, we show that the transcription factor E2F4 directly binds to the USP10 promoter, activating its expression in HNSCC cells. Importantly, we uncover that RAF265-a compound already approved by the FDA, effectively targets USP10, leading to decreased SCD1 levels. This results in suppressed tumor growth and increased ferroptosis in both cellular and animal models of HNSCC. Taken together, our findings highlight the pivotal function of the E2F4-USP10-SCD1 signaling axis in regulating ferroptosis and tumor progression in HNSCC, suggesting that pharmacological inhibition of USP10 via RAF265 may represent a viable therapeutic strategy for treating this aggressive cancer.

## Results

### USP10 associates with SCD1 and enhances its protein levels

**Fig. 1:**
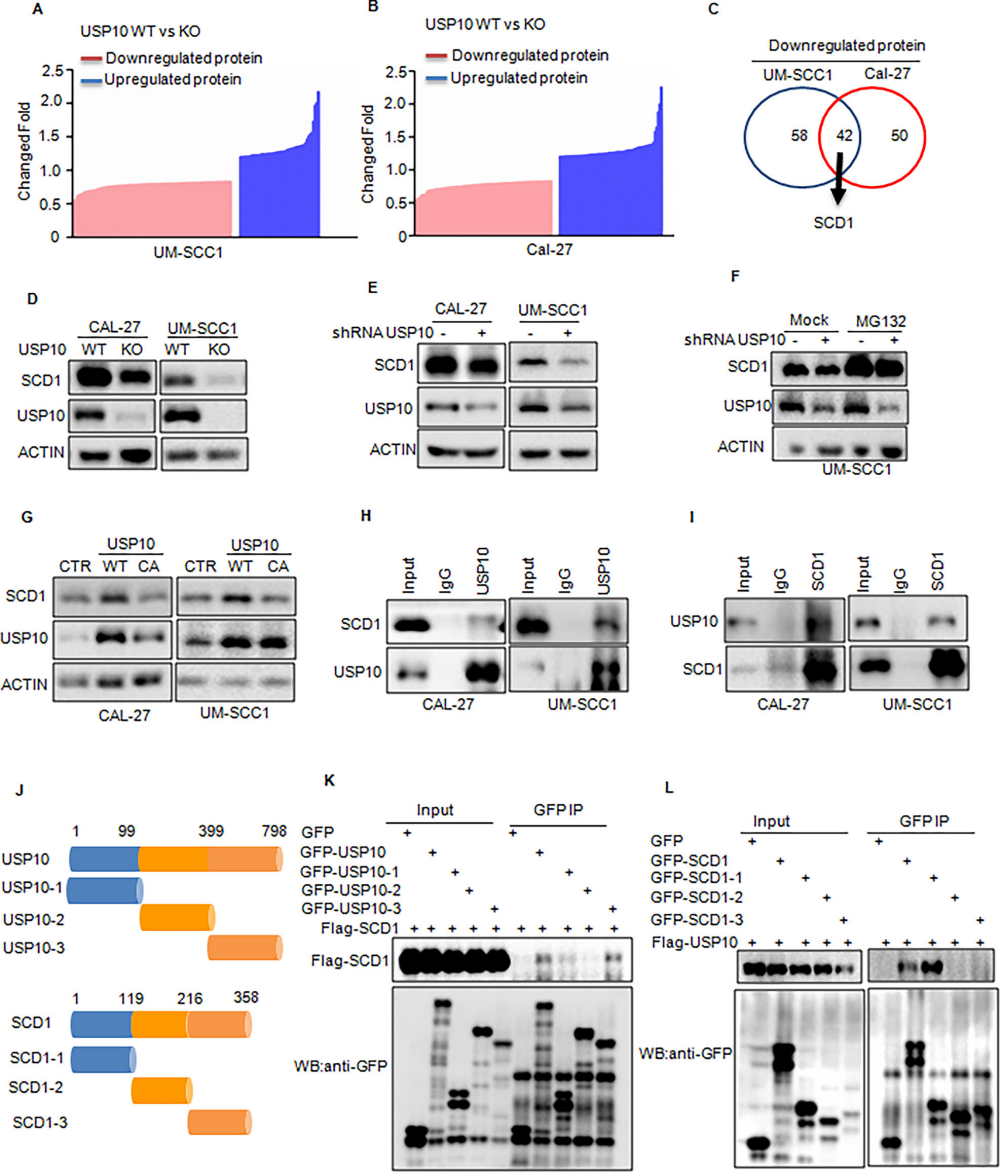
USP10 binds to SCD1 and promotes its expression in HNSCC cells. **A, B** Label-free quantitative proteomics analysis was performed to identify differentially expressed proteins in UM-SCC1 and CAL-27 cells with or without USP10 knockout. **C** Venn diagrams illustrate overlapping differentially regulated genes in both USP10-deficient cell lines. **D** Western blot was employed to assess SCD1 protein levels in UM-SCC1 and CAL-27 cells under USP10 knockout conditions. **E** The expression of SCD1 in the specified cell lines was examined via western blot. **F** UM-SCC1 cells, with or without USP10 knockdown, were exposed to MG132 for 8 h, followed by western blot analysis of SCD1 expression. **G** Wild-type USP10 or the CA mutant was overexpressed in UM-SCC1 and CAL-27 cells, and subsequent changes in SCD1 protein levels were evaluated. **H, I** Immunoprecipitation assays were carried out on lysates from UM-SCC1 and CAL-27 cells using control IgG, anti-USP10, or anti-SCD1 antibodies, followed by immunoblotting with the indicated detection antibodies. **J–L** Schematic representations of USP10 and SCD1 domain structures are shown. The corresponding plasmid constructs were transfected into 293 T cells, and after 24 h, co-immunoprecipitation experiments were performed using anti-Flag or anti-GFP antibodies.

To explore the molecular mechanisms through which USP10 contributes to HNSCC progression, we performed label-free quantitative proteomic analysis on UM-SCC1 and CAL-27 cells with or without USP10 knockout (Fig. 1A, B). This approach identified 42 proteins that were downregulated upon USP10 loss, among which stearoyl-CoA desaturase 1 (SCD1) exhibited a marked reduction (Fig. 1C).

To validate the connection between USP10 and SCD1, we examined SCD1 expression in both USP10-knockout and knockdown HNSCC cell lines. Consistent with the proteomics results, depletion of USP10 led to decreased SCD1 protein levels (Fig. 1D, E). Additionally, pharmacological inhibition of USP10 using spautin-1 significantly suppressed SCD1 expression (Supplementary Fig. 1A). To determine whether this regulatory effect depends on the deubiquitinase activity of USP10, we treated USP10-deficient cells with MG132, a proteasome inhibitor, and observed that MG132 rescued the decline in SCD1 caused by USP10 silencing (Fig. 1F). Moreover, overexpression of wild-type USP10, but not the catalytically inactive CA mutant elevated SCD1 levels in HNSCC cells, confirming that USP10 stabilizes SCD1 in a deubiquitination-dependent manner (Fig. 1G).

Given that USP10 typically interacts physically with its substrates to exert its function, we next assessed whether USP10 binds directly to SCD1. Coimmunoprecipitation experiments revealed that exogenously expressed GFP-tagged SCD1 co-precipitated with FLAG-tagged USP10, and reciprocally, FLAG-USP10 was detected in GFP-SCD1 immunoprecipitates (Supplementary Fig. 1B, C). Importantly, an interaction between endogenous USP10 and SCD1 was also confirmed in HNSCC cells (Fig. 1H, I).

To define the specific domains responsible for their interaction, we co-expressed Flag-tagged SCD1 with various GFP-tagged fragments of USP10 in HEK293T cells. Co-IP assays showed that the C-terminal region of USP10 (amino acids 399–789) is essential for binding SCD1. Conversely, mapping the SCD1 side revealed that its N-terminal domain (amino acids 1–119) mediates the association with USP10 (Fig. 1J–L). Collectively, these findings confirm that USP10 directly interacts with SCD1 and positively regulates its expression in HNSCC.

### USP10 stabilizes SCD1 by removing K48-linked ubiquitin chains

**Fig. 2:**
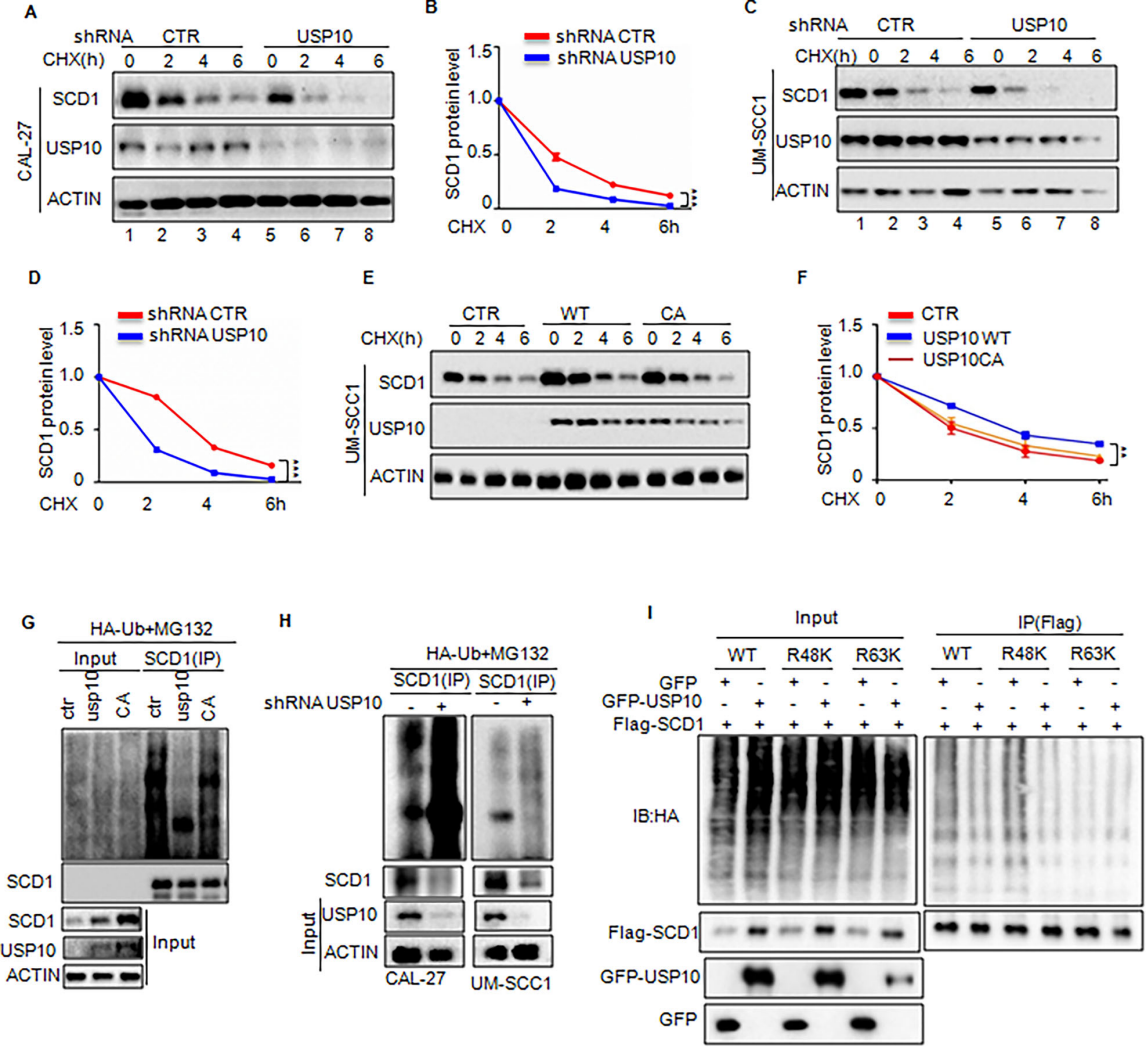
USP10 stabilizes SCD1 by inhibiting its degradation. **A–D** UM-SCC1 and CAL-27 cells, with or without USP10 knockdown, were treated with cycloheximide (CHX, 10 mg/mL) for various durations to determine the half-life of SCD1. **E, F** Overexpression of wild-type USP10 or the CA mutant in UM-SCC1 cells was used to evaluate the impact on SCD1 protein stability. **G** HA-Ub was co-expressed with or without wild-type or mutant USP10 in UM-SCC1 cells, followed by MG132 treatment and immunoprecipitation with SCD1 antibody to detect ubiquitinated SCD1. **H** In UM-SCC1 and CAL-27 cells with or without USP10 knockdown, HA-Ub was transfected, cells were treated with MG132 for 8 h, and SCD1 ubiquitination was analyzed by immunoprecipitation and Western blot. **I** HEK293T cells were transfected with HA-Ub (wild-type or R48K/R63K mutants), Flag-SCD1, and either GFP-USP10 or GFP control; immunoprecipitation was used to analyze the types of polyubiquitin chains on SCD1 that are removed by USP10. Data in **B**, **D**, **F** were representative results of three independent experiments and analyzed using Student’s *t* test; *p < 0.05, **p < 0.01, ***p < 0.001.

To evaluate how USP10 influences SCD1 stability, we conducted cycloheximide (CHX) chase assays in USP10-depleted HNSCC cells. Western blot analysis demonstrated that knockdown of USP10 accelerated SCD1 degradation, indicating reduced protein stability (Fig. 2A–D). In contrast, ectopic expression of wild-type USP10, but not the CA mutant, prolonged the half-life of SCD1 (Fig. 2E, F), underscoring the necessity of catalytic activity in this process.

Further experiments showed that overexpression of functional USP10—yet not the inactive mutant—significantly reduced polyubiquitination of SCD1 (Fig. 2G), whereas suppression of USP10 increased its ubiquitination levels (Fig. 2H). Then, we investigated the type of ubiquitin linkage targeted by USP10 and found that USP10 specifically removes K48-linked ubiquitin chains from SCD1, with no significant effect on K63-linked chains (Fig. 2I). These results collectively demonstrate that USP10 enhances SCD1 stability by cleaving K48-linked ubiquitin moieties, thereby preventing proteasomal degradation and maintaining high SCD1 levels in HNSCC cells.

### USP10 attenuates RSL3-triggered ferroptosis through upregulation of SCD1

**Fig. 3:**
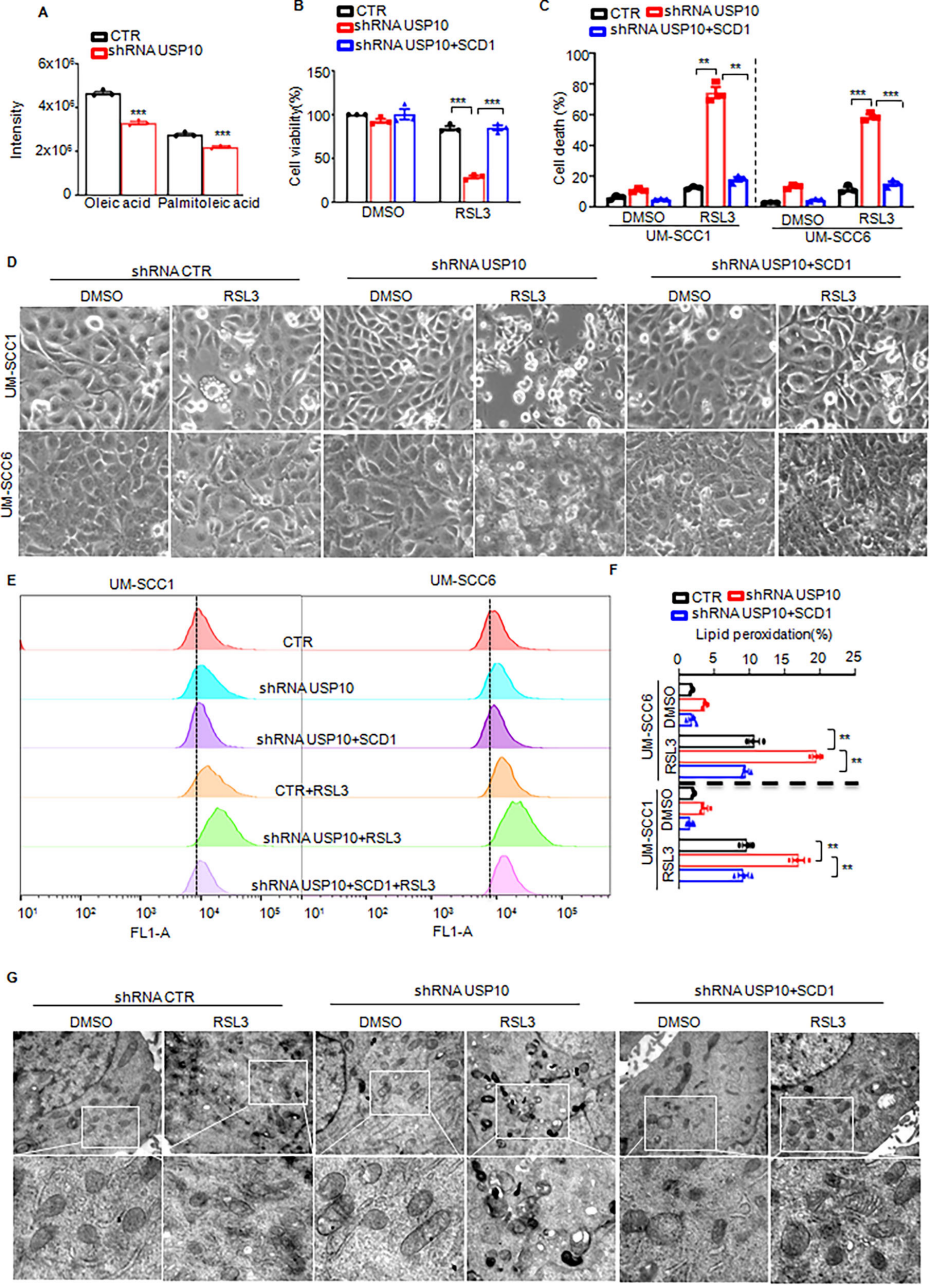
USP10 alleviates ferroptosis by enhancing SCD1 expression. **A** The levels of oleic acid and palmitoleic acid were determined by lipidomic mass spectrometry in USP10-depleting cells. **B** SCD1 was overexpressed in USP10-depleted UM-SCC1 cells, which were then treated with RSL3, and cell survival was measured. **C, D** Representative phase-contrast microscopy images show morphological changes and cell death in the indicated groups after RSL3 treatment. **E, F** Lipid peroxidation levels were quantified by flow cytometry using C11-BODIPY staining in the specified cell lines with or without RSL3 exposure. **G** TEM analysis was performed on USP10-depleted UM-SCC1 cells overexpressing SCD1 and treated with RSL3. Data in **A, B, C, F** were representative results of three independent experiments and analyzed using Student’s *t* test; *p < 0.05, **p < 0.01, ***p < 0.001.

A growing body of research has demonstrated that SCD1 plays a key role in the de novo production of monounsaturated fatty acids, specifically converting stearic acid and palmitic acid into their respective D9-cis isomers—oleic acid (OA) and palmitoleic acid—thereby inhibiting ferroptosis [22]. Given this, we sought to determine whether USP10 influences de novo production of monounsaturated fatty acids and ferroptosis in HNSCC by stabilizing SCD1. To test this, we first investigated the effect of USP10 on the oleic acid (OA) and palmitoleic acid, two main lipid products of SCD1. As expected, knockdown of USP10 reduced the levels of oleic acid and palmitoleic acid, with a more significant decrease in oleic acid (Fig. 3A). Then, we evaluated the impact of USP10 on ferroptosis and induced ferroptosis using RSL3 in control and USP10-knockdown HNSCC cells. Depletion of USP10 significantly heightened RSL3-induced cytotoxicity, an effect that was effectively reversed by ferrostatin-1 (Ferr-1), a specific ferroptosis inhibitor. In contrast, neither the apoptosis inhibitor Z-VAD-FMK nor the autophagy inhibitor 3-methyladenine (3-MA) could rescue cell viability under these conditions (Supplementary Fig. 2A). Next, we restored SCD1 expression in USP10-deficient cells and observed that re-expression of SCD1 counteracted the reduction in cell viability and the increase in cell death caused by RSL3 treatment (Fig. 3B–D and Supplementary Fig. 2B). Furthermore, elevated SCD1 levels mitigated the accumulation of lipid peroxidation in USP10-depleted UM-SCC1 and UM-SCC6 cells upon RSL3 exposure (Fig. 3E, F). Transmission electron microscopy (TEM) analysis revealed that USP10 knockdown resulted in characteristic ferroptotic morphological changes—such as mitochondrial condensation and increased membrane density—which were largely prevented when SCD1 was overexpressed (Fig. 3G). These findings collectively demonstrate that USP10 protects HNSCC cells from RSL3-induced ferroptosis by enhancing SCD1 expression.

### USP10 drives HNSCC progression via SCD1 upregulation

**Fig. 4:**
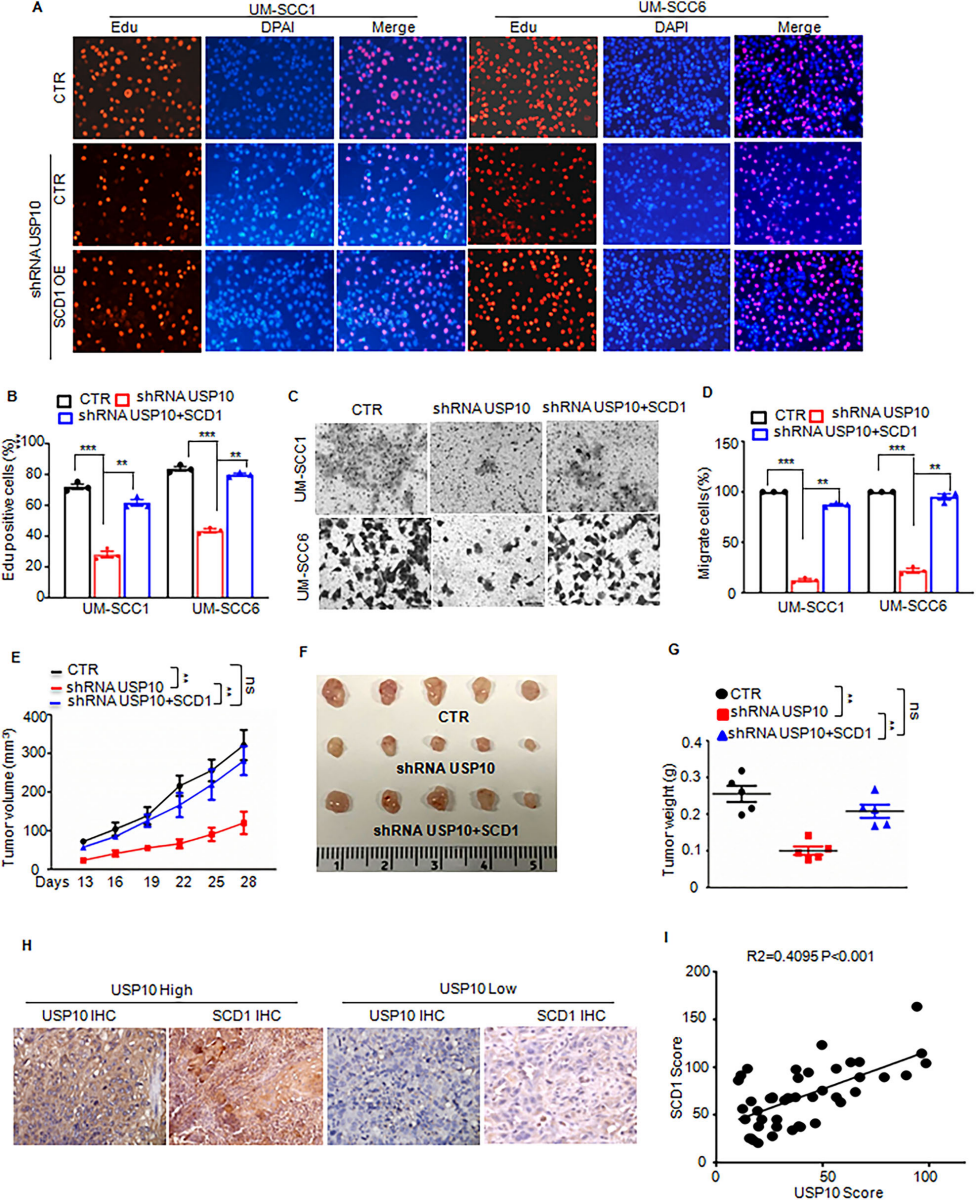
USP10 enhances tumor cell proliferation and migration through upregulation of SCD1. **A, B** Cell proliferation was assessed using EdU incorporation assays. **C, D** Transwell migration assays were used to evaluate cell motility. Indicated UM-SCC1 cells were subcutaneously injected into female NOD/SCID mice (*n* = 5). Representative images of xenograft tumors are shown, along with tumor volume (**E**) and weight (**G**) measurements. **H, I** Immunohistochemical analysis of clinical HNSCC tissue samples (*n* = 45) was conducted using USP10 and SCD1 antibodies to explore the correlation between their expression levels. Data in **A, B, E, F, H** were representative results of three independent experiments and analyzed using Student’s *t* test; *p < 0.05, **p < 0.01, ***p < 0.001.

While SCD1 has been implicated in the development of various cancers, its role in HNSCC remains poorly defined [23]. We therefore evaluated the functional impact of SCD1 on HNSCC cell proliferation and migration. Silencing SCD1 markedly impaired both cellular growth and migratory capacity (Supplementary Fig. 3A–E). Notably, ectopic expression of SCD1 rescued the proliferative and migratory defects in USP10-deficient HNSCC cells, as confirmed by EdU incorporation and Transwell assays (Fig. 4A–D). In vivo xenograft experiments further showed that the impaired tumour formation caused by USP10 depletion was restored upon SCD1 re-expression (Fig. 4E–G). Immunohistochemical staining for 4-hydroxynonenal (4HNE), a marker of lipid peroxidation, revealed increased positive signals in tumours derived from USP10-silenced cells, indicating elevated oxidative damage; however, this effect was abrogated when SCD1 was overexpressed (Supplementary Fig. 3F).

To further validate the clinical relevance of the USP10/SCD1 axis, we performed immunohistochemical analysis and correlation assessment in HNSCC tissue samples. The results showed that SCD1 is upregulated in HNSCC tissues and positively correlates with USP10 expression levels (Fig. 4J, K and Supplementary Fig. 3G, H). Together, these data support a model in which USP10 promotes HNSCC malignancy by elevating SCD1 expression.

### E2F4 directly activates USP10 transcription by binding to its promoter in HNSCC

**Fig. 5:**
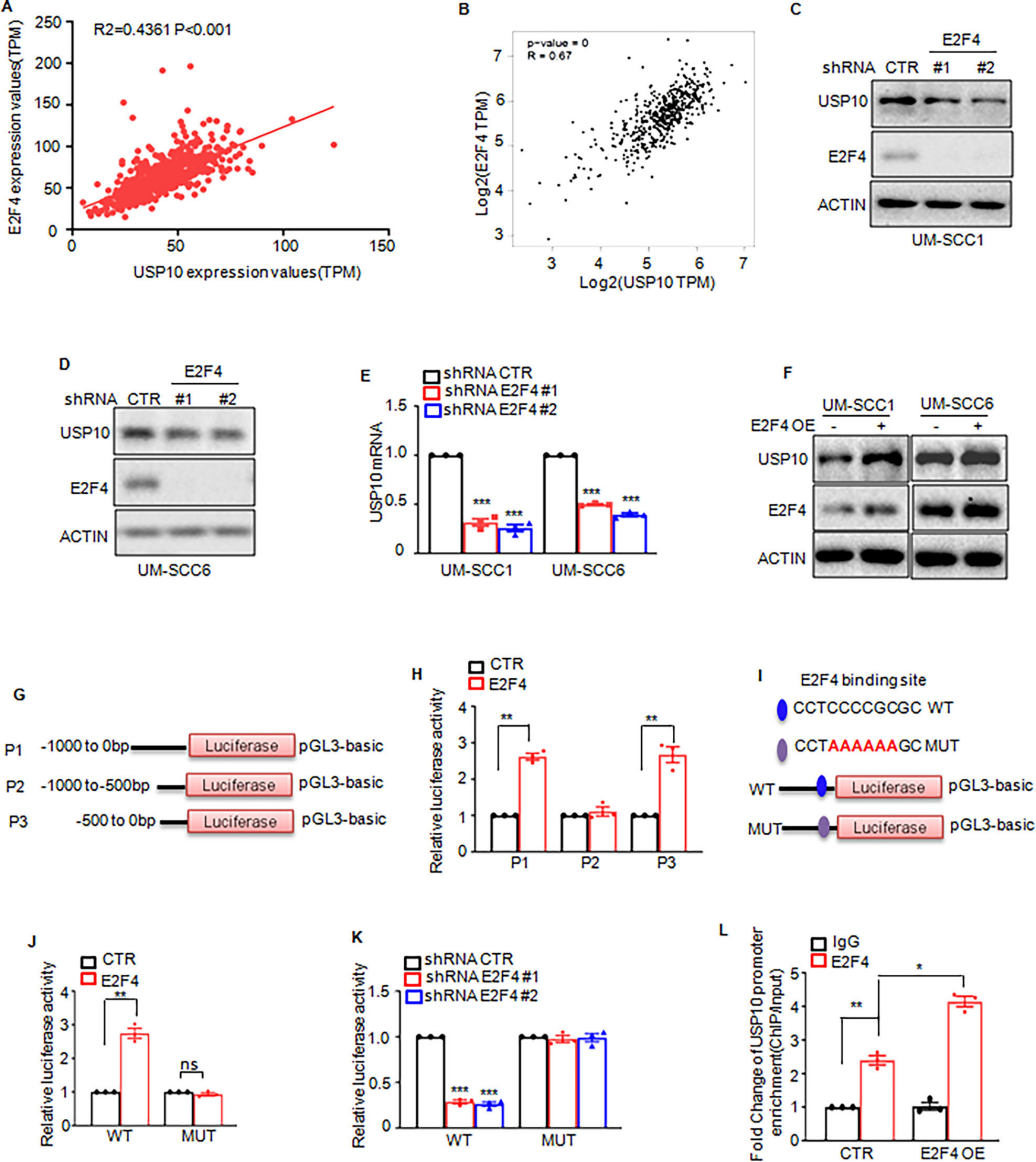
E2F4 transcriptionally activates USP10 expression in HNSCC cells. **A, B** The relationship between E2F4 and USP10 expression in head and neck squamous cell carcinoma (HNSCC) was evaluated using data from the UALCAN and GEPIA databases. **C–E** Western blot and qRT-PCR analyses were performed to determine USP10 expression levels in HNSCC cells following E2F4 knockdown. **F** The impact of E2F4 overexpression on USP10 protein levels was assessed using Western blot. **G** Schematic representation of pGL3-based reporter constructs (P1, P2, P3) containing different regions of the USP10 promoter, used in luciferase assays to evaluate promoter activity. **H** UM-SCC1 cells were co-transfected with P1/2/3 reporter plasmids and Renilla luciferase vector, with or without E2F4 overexpression, followed by measurement of luciferase activity to assess USP10 promoter activation. **I** Potential E2F4 binding sites within the USP10 promoter were predicted using the JASPAR database; wild-type and mutant versions of the binding site were cloned into the pGL3 vector for functional validation. **J** Wild-type (WT) or mutant (MUT) USP10 promoter reporters along with Renilla control were transfected into UM-SCC1 cells with or without E2F4 overexpression, and luciferase activity was measured. **K** Similar transfection experiments were conducted in the presence or absence of E2F4 knockdown to further confirm regulatory specificity. **L** Chromatin immunoprecipitation (ChIP) assays were performed in UM-SCC1 cells to validate the direct binding of E2F4 to the USP10 promoter, with or without E2F4 overexpression. Data in **E, H, J, K, L** were representative results of three independent experiments and analyzed using Student’s *t* test; *p < 0.05, **p < 0.01, ***p < 0.001.

To uncover the mechanism underlying elevated USP10 mRNA levels in HNSCC, we analyzed genes co-expressed with USP10 using the UALCAN database. Among the top 25 correlated genes, two transcription factors—E2F4 and YY1—were identified (Fig. 5A, B and Supplementary Fig. 4A–C). Functional validation via siRNA-mediated knockdown revealed that silencing E2F4, but not YY1, significantly reduced USP10 expression in HNSCC cells (Fig. 5C–E and Supplementary Fig. 4D–F), whereas overexpression of E2F4 enhanced USP10 levels (Fig. 5F).

To investigate whether E2F4 regulates USP10 at the transcriptional level, we cloned different fragments of the USP10 promoter into the pGL3-basic luciferase vector, generating constructs P1, P2, and P3 (Fig. 5G). Co-transfection of these reporters with an E2F4 expression plasmid into HNSCC cells showed that E2F4 overexpression increased luciferase activity in P1 and P3, but not in P2, suggesting that the region between −500 and 0 bp (P3) is crucial for E2F4-mediated transactivation (Fig. 5H). Using the JASPAR database, we predicted a putative E2F4-binding site within the P3 region. To confirm its functionality, we generated two reporter constructs: one containing the wild-type E2F4-binding sequence and another with a mutated version (Fig. 5I). Luciferase assays demonstrated that modulation of E2F4 expression (overexpression or knockdown) altered the activity of the wild-type promoter but had no effect on the mutant (Fig. 5J, K).

Chromatin immunoprecipitation (ChIP) assays further validated direct binding of E2F4 to the endogenous USP10 promoter region harboring the predicted site; the binding capacity of E2F4 for the USP10 promoter was elevated in E2F4-overexpressing HNSCC cells (Fig. 5L). In summary, our data suggest that E2F4 acts as a transcriptional activator of USP10 in HNSCC cells.

### E2F4 drives tumor aggressiveness and SCD1 expression by activating USP10

**Fig. 6:**
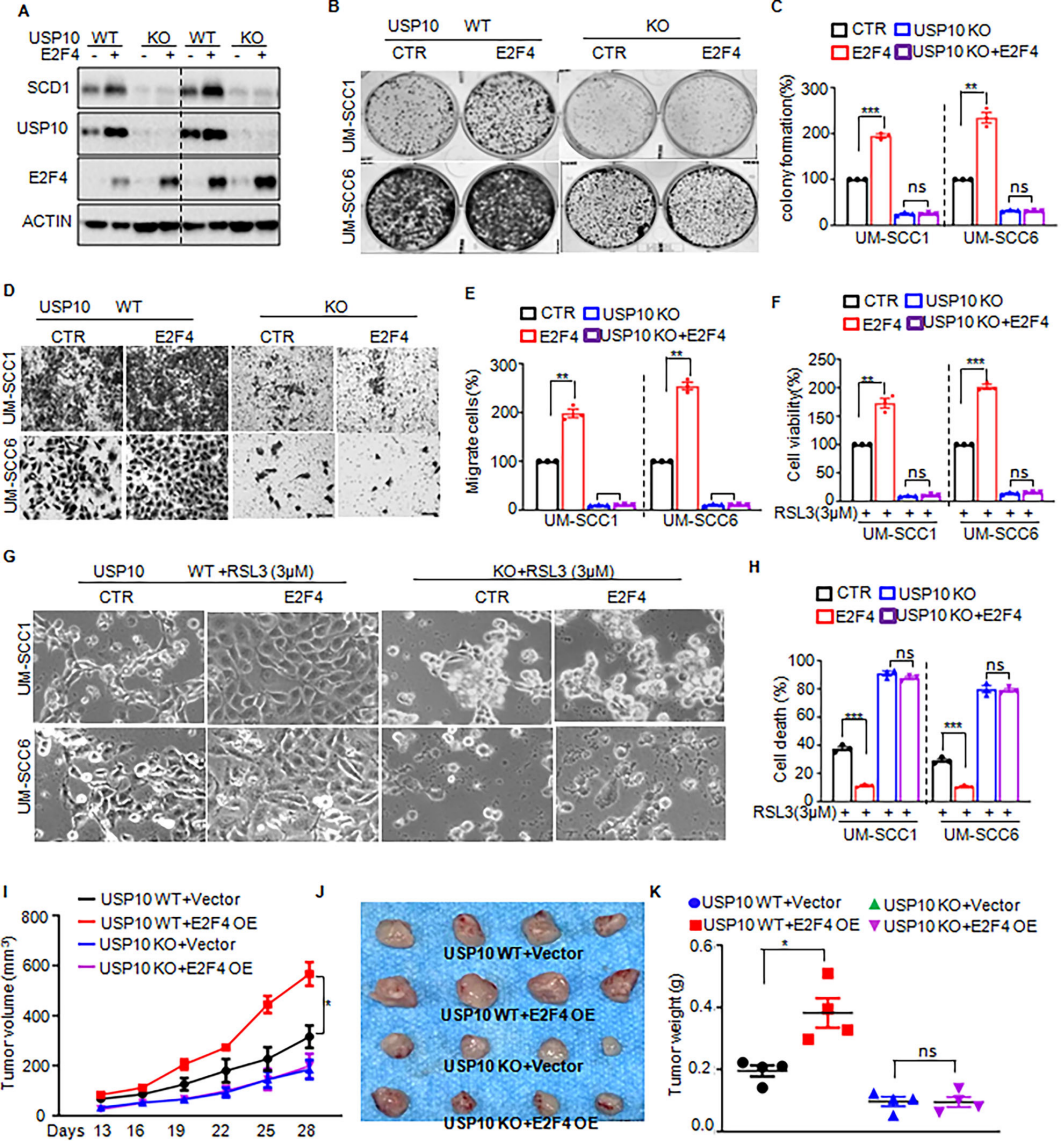
E2F4 drives HNSCC progression and enhances SCD1 expression via upregulation of USP10. **A** E2F4 was overexpressed in UM-SCC1 and UM-SCC6 cells with or without USP10 knockout, and SCD1 protein levels were examined by western blot. **B–E** Colony formation and Transwell migration assays were employed to evaluate the effects of E2F4 on proliferative and migratory capacities in both USP10 wild-type and knockout cells. **F–H** Cells expressing E2F4 with or without USP10 deletion were treated with RSL3, and cell viability was assessed using MTT assays, while morphological changes and cell death were visualized by phase-contrast microscopy. **I–K** Indicated UM-SCC1 cells were implanted subcutaneously into female NOD/SCID mice (*n* = 4). Representative images of xenograft tumors are shown, along with tumor volume (**I**) and tumor weight (**K**) measurements. Data in **C, E, F**,**p < 0.01, ***p < 0.001.

To determine whether E2F4 enhances the malignant behavior of HNSCC and upregulates SCD1 through USP10, we ectopically expressed E2F4 in UM-SCC1 and UM-SCC6 cells, both in the presence and absence of USP10. In wild-type cells, forced expression of E2F4 led to a significant increase in SCD1 levels. However, this induction was completely lost upon USP10 knockout (Fig. 6A). Similarly, the pro-tumorigenic effects of E2F4—such as enhanced cell proliferation, migration, and resistance to ferroptosis—were eliminated when USP10 was absent (Fig. 6B–H). In vivo xenograft experiments further confirmed that the tumor-promoting capacity of E2F4 was nullified in USP10-deficient conditions (Fig. 6I–K). These findings collectively indicate that USP10 is essential for mediating E2F4-induced oncogenic progression and SCD1 upregulation, highlighting a critical E2F4-USP10-SCD1 regulatory axis in HNSCC.

### RAF265 interacts with USP10 to facilitate SCD1 degradation

**Fig. 7:**
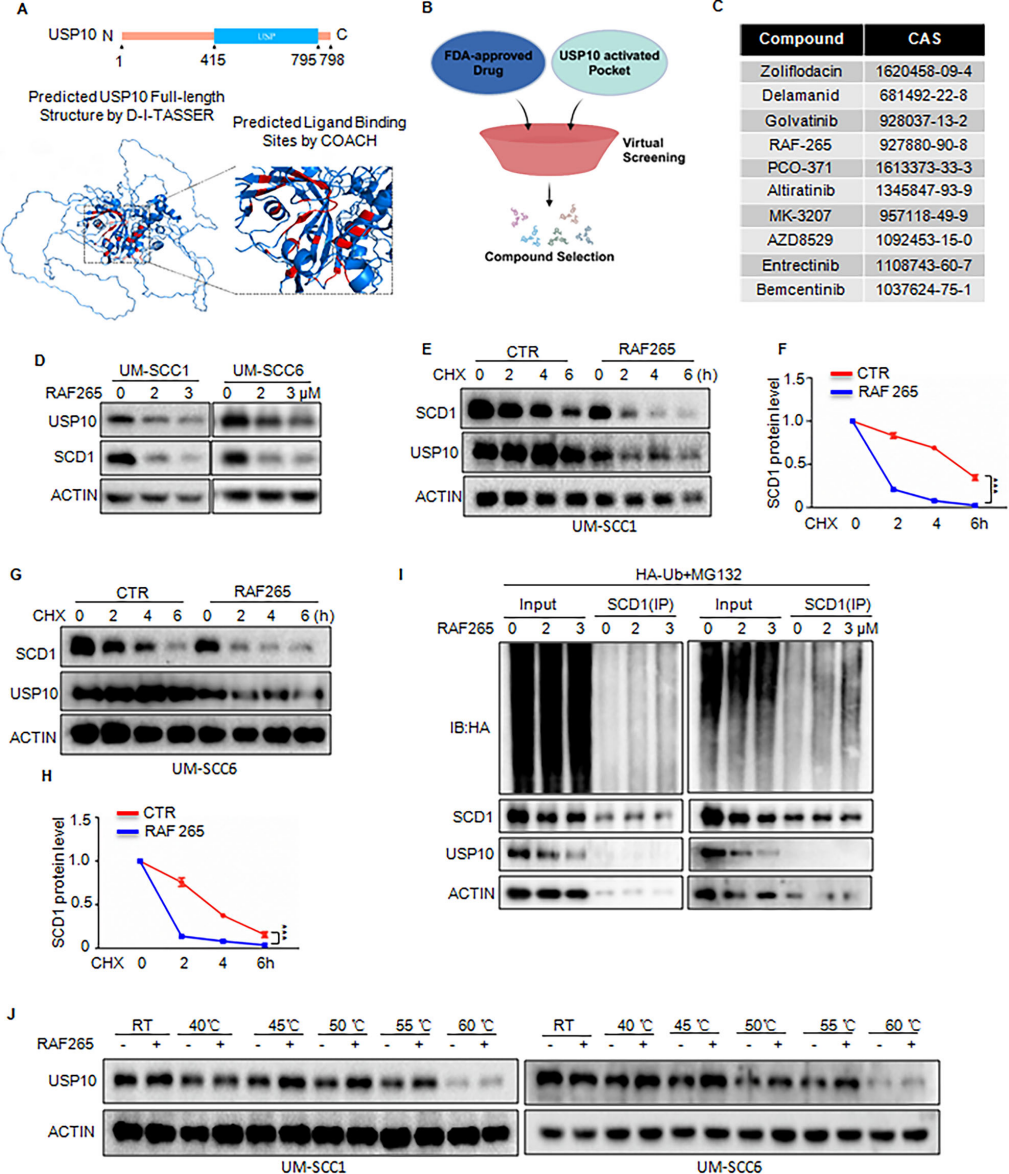
RAF265 directly targets USP10. **A** Schematic representation of human USP10, highlighting the ubiquitin-specific protease (USP) domain. The predicted full-length three-dimensional structure of USP10 was generated using D-I-TASSER, displayed as a blue cartoon model. Potential ligand-binding sites, identified by COACH, are marked in red. **B**, **C** In silico screening of FDA-approved drugs was conducted to discover compounds capable of binding to USP10; the top 10 candidate molecules are listed. **D** Western blot analysis was used to examine protein levels of SCD1 and USP10 in HNSCC cells treated with 0, 2, or 3 µM RAF265 for 24 h. **E**–**G** UM-SCC1 and UM-SCC6 cells were pre-treated with or without 3 µM RAF265 for 24 h, followed by exposure to cycloheximide (CHX, 10 µg/mL) for various durations to assess the stability and half-life of SCD1. **H** HA-tagged ubiquitin was transfected into UM-SCC1 and UM-SCC6 cells under specified RAF265 treatment conditions. Cells were then treated with MG132 for 8 h, lysates were immunoprecipitated with anti-SCD1 antibody, and SCD1 ubiquitination levels were detected by Western blot. **I** Cellular Thermal Shift Assay (CETSA) demonstrated that RAF265 stabilizes USP10 against heat-induced denaturation, indicating direct target engagement. **J** Cellular Thermal Shift Assay showing that RNF265 attenuated temperature-induced degeneration of the USP10 protein in UM-SCC1 and UM-SCC6 cells. Data in (**F**, **H**) were representative results of three independent experiments and analyzed using Student’s *t* test; *p < 0.05, **p < 0.01, ***p < 0.001.

Given the critical role of USP10 in enhancing SCD1 stability and driving HNSCC progression, we hypothesized that targeting USP10 could represent a promising therapeutic strategy for HNSCC. To test this, we first predicted the full-length three-dimensional structure of USP10 using D-I-TASSER. Guided by this model, potential catalytic sites within USP10 were identified through COACH analysis (Fig. 7A). We then conducted virtual screening of FDA-approved compounds to discover molecules capable of binding these active pockets. Based on docking scores, ten candidates with binding energies ≤−10.0 kcal/mol were selected and evaluated for their impact on USP10 and SCD1 expression levels (Fig. 7B, C). Among them, RAF265 emerged as a potent suppressor of both USP10 and SCD1 in HNSCC cells (Fig.7D and Supplementary Fig. 5A, B). Notably, further experiments revealed that RAF265 decreased SCD1 protein stability while increasing its ubiquitination in a dose-dependent manner(Fig. 7E–I).

To validate the physical interaction between RAF265 and USP10, Cellular Thermal Shift Assay (CETSA) was performed. HNSCC cells treated with or without RAF265 were subjected to controlled heat stress. As anticipated, USP10 underwent rapid denaturation under thermal challenge; however, in RAF265-treated cells, USP10 exhibited enhanced thermal stability at elevated temperatures, suggesting direct engagement between RAF265 and USP10 (Fig. 7J). Collectively, these findings confirm that RAF265 functions as a small-molecule inhibitor of USP10 and effectively blocks the USP10/SCD1 signaling axis.

### RAF265 suppresses malignant phenotypes of HNSCC and induces ferroptosis in vitro and in vivo

**Fig. 8:**
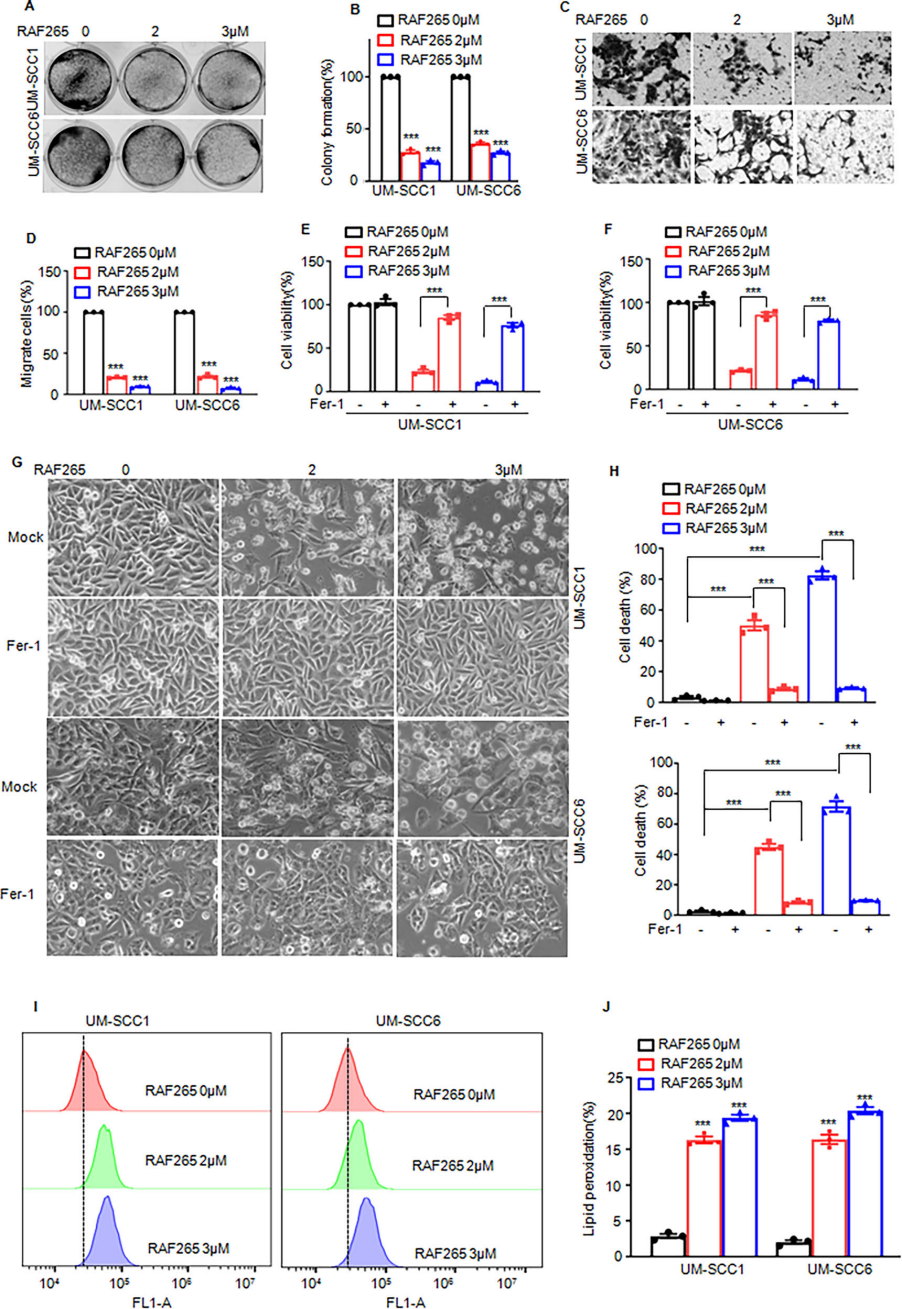
RAF265 inhibits HNSCC cell proliferation and migration while promoting ferroptosis. **A–D** UM-SCC1 and UM-SCC6 cells were treated with 0, 2, or 3 µM RAF265, and cell growth and migratory capacity were evaluated using colony formation and Transwell assays, respectively. **E–G** Cells were exposed to RAF265 alone or in combination with Ferrostatin-1 (Ferr-1), followed by assessment of cell viability and death to confirm ferroptotic mechanisms. **H, I** Flow cytometry analysis of C11-BODIPY-stained cells was performed to measure lipid peroxidation levels in UM-SCC1 and UM-SCC6 cells with or without RAF265 treatment. Data in **B, D, E, F, H, J** were representative results of three independent experiments and analyzed using Student’s *t* test; *p < 0.05, **p < 0.01, ***p < 0.001.

To assess the therapeutic efficacy of RAF265 in HNSCC, UM-SCC, UM-SCC6, and Cal-27 cell lines were exposed to increasing concentrations (0, 2, and 3 μM) of RAF265. Treatment resulted in significant inhibition of cell proliferation and migration(Fig. 8A–D and Supplementary Fig. 6A–C). Next, we investigated whether RAF265 triggers ferroptosis in HNSCC cells. As shown in Fig. 8E–H, RAF265 markedly reduced cell viability and induced cell death—effects that were rescued by co-treatment with ferrostatin-1 (Ferr-1), a specific ferroptosis inhibitor—indicating that RAF265 exerts its cytotoxic effects primarily through induction of ferroptosis. Moreover, RAF265 treatment led to a pronounced increase in lipid peroxidation, a hallmark of ferroptotic cell death(Fig. 8I, J).

**Fig. 9:**
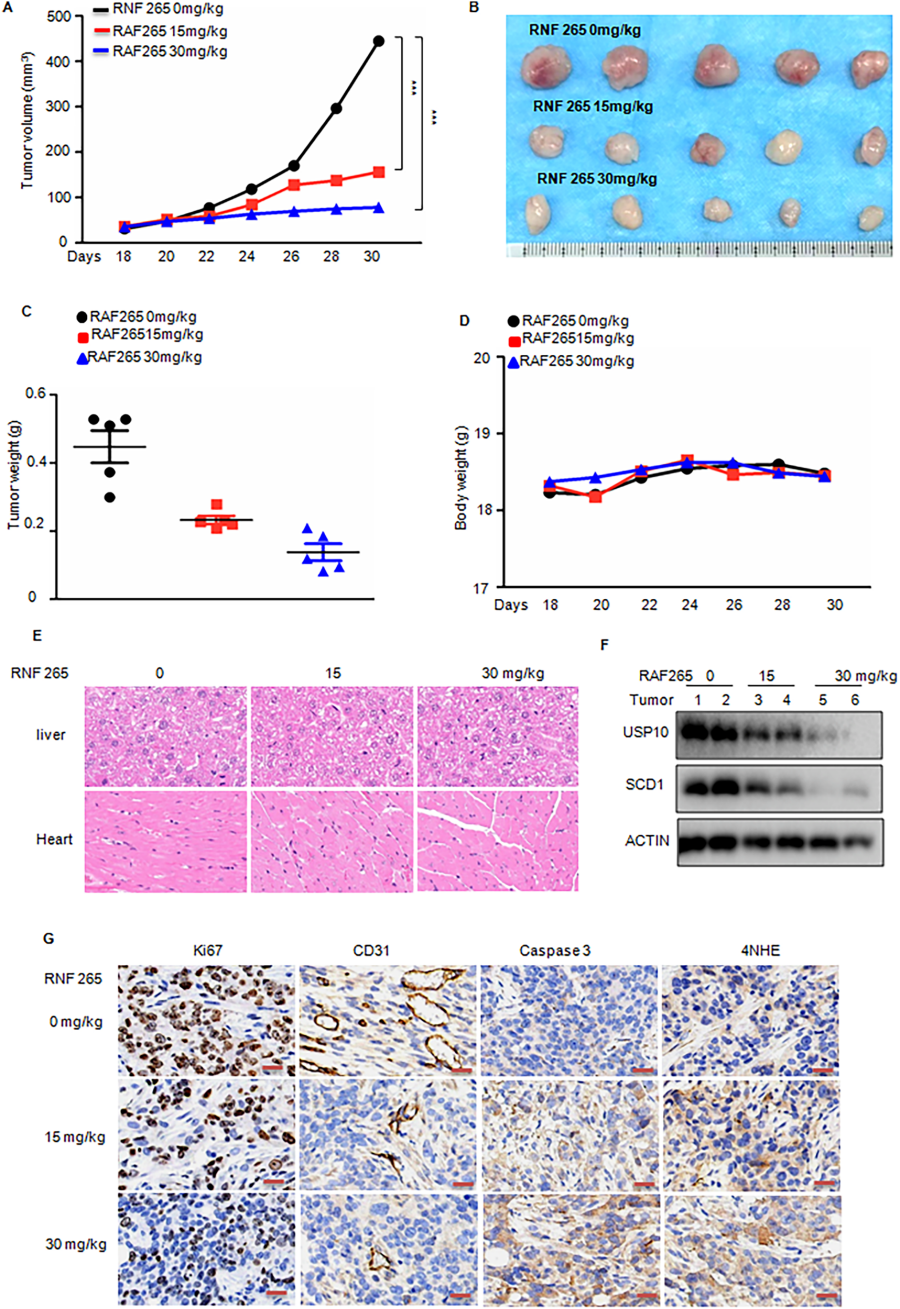
RAF265 suppresses tumor growth of UM-SCC1 cells in a xenograft mouse model. **A–C** UM-SCC1 cells were subcutaneously injected into female NOD/SCID mice (*n* = 5). Starting two weeks post-implantation, mice were orally administered RAF265 at doses of 15 or 30 mg/kg/day for 16 days before tumor excision. Representative images of excised tumors are shown, along with tumor volume (**A**) and weight (**C**) measurements. **D** Changes in mouse body weight were monitored throughout the treatment period. **E** Hematoxylin and eosin (H&E) staining was performed on heart and liver tissues to evaluate potential drug-induced toxicity. **F** Western blot was used to detect USP10 and SCD1 expression levels in tumor tissues. **G** Immunohistochemical (IHC) staining of tumor sections was carried out to assess expression of Ki-67, CD31, Caspase-3 and 4NHE. Scale bar: 20 μm. Statistical analysis for **A, C, D** was performed using Student’s t-test; *p < 0.05, **p < 0.01, ***p < 0.001.

We further examined the anti-tumor activity of RAF265 in vivo using a UM-SCC1 xenograft mouse model. Administration of RAF265 significantly inhibited tumor growth, as evidenced by reduced tumor volume and weight compared to control animals (Fig. 9A–C). Importantly, no significant changes in body weight or histopathological features of liver and heart tissues were observed in treated mice relative to saline controls, suggesting minimal systemic toxicity (Fig. 9D, E). Western blot and immunohistochemical analyses of tumor tissues from RAF265-treated mice showed marked downregulation of USP10 and SCD1 (Fig. 9F), along with decreased expression of Ki-67 and CD31 (markers of proliferation and angiogenesis), and increased caspase-3-positive cells (indicative of apoptosis) (Fig. 9G). These results demonstrate that RAF265 effectively impedes tumor progression while promoting ferroptotic cell death, highlighting its potential as a safe and effective agent against HNSCC.

## Discussion

In this study, we demonstrate that USP10 functions as an oncogenic driver in head and neck squamous cell carcinoma (HNSCC) by acting as the first identified deubiquitinating enzyme for SCD1. USP10 physically interacts with SCD1 and prevents its proteasomal degradation, thereby enhancing SCD1 protein levels. This stabilization of SCD1 contributes to the progression of HNSCC and confers resistance to ferroptosis. Furthermore, our findings reveal that the transcription factor E2F4 directly binds to the USP10 promoter, activating its expression in HNSCC.

As a member of the ubiquitin-specific protease family, USP10 has been reported to exert context-dependent roles in cancer development [11]. It acts as a tumor suppressor in lung cancer by stabilizing KLF4 and PTEN. [18, 21]. Conversely, in several other malignancies, USP10 promotes tumorigenesis. Recent studies have highlighted its involvement in HNSCC, where it enhances POLR2A expression and inhibits ferroptosis. Additionally, USP10 stabilizes BAZ1A to drive tumor progression in HNSCC.

To further investigate the tumour-promoting mechanism of USP10, label-free quantitative proteomics was performed and identified that loss of USP10 downregulated SCD1 expression. SCD1 is a key enzyme for the de novo synthesis of fatty acids and catalyses the conversion of saturated fatty acids into monounsaturated fatty acids.

To gain deeper insights into the pro-tumorigenic function of USP10, we conducted label-free quantitative proteomic analysis, which revealed that depletion of USP10 leads to reduced expression of SCD1. SCD1 is a critical enzyme involved in the biosynthesis of monounsaturated fatty acids from saturated fatty acids [24]. Elevated SCD1 levels are observed in various cancers and are linked to poor clinical outcomes [23]. Current research on SCD1 regulation has primarily focused on transcriptional control mechanisms. For instance, SREBP1, a master regulator of lipid metabolism, activates SCD1 transcription [25], whereas the tumor suppressor p53 represses it by binding to the SCD1 promoter [26]. HOXB9 has also been shown to transcriptionally upregulate SCD1 and protect melanoma cells from brusatol-induced death [27].

Notably, recent work by Liu et al. demonstrated that CirPKN2 induces ferroptosis in bladder cancer by promoting SCD1 ubiquitination, identifying STUB1 as the E3 ubiquitin ligase responsible for this modification [28]. These findings collectively suggest that SCD1 expression is regulated through ubiquitin-proteasome pathways. In this study, we identify USP10 as a novel deubiquitinase of SCD1 that specifically removes K48-linked polyubiquitin chains, thus preventing its degradation and promoting its accumulation in HNSCC.

Accumulating evidence supports a crucial role for SCD1 in suppressing ferroptosis by promoting the synthesis of monounsaturated fatty acids (MUFA), modulating the composition of cell membrane lipids, and reducing lipid peroxidation [22]. In addition, SCD1 was reported to promote tumor growth across multiple cancer types, including lung, colorectal, pancreatic, breast, and melanoma [29–33]. However, its functional significance in HNSCC remains largely unexplored. Our results show that SCD1 is overexpressed in HNSCC tissues and significantly enhances cancer cell proliferation and migration. Importantly, we demonstrate that USP10 exerts its oncogenic effects—at least in part—by upregulating SCD1, leading to suppression of ferroptosis and accelerated tumor progression.

We further explored the regulatory mechanism underlying elevated USP10 expression at the RNA level in HNSCC and identified E2F4 as a novel transcriptional activator of USP10. E2F4 belongs to the E2F family of transcription factors, which are key regulators of cell cycle progression and tumorigenesis [34–37]. It is frequently overexpressed in HNSCC and correlates with poor prognosis and immune infiltration patterns [38–40]. Our data confirm the oncogenic role of E2F4 in HNSCC, showing that it drives tumor development and inhibits ferroptosis by activating the USP10/SCD1 signaling axis.

Currently, targeting the ubiquitination pathway has gained increasing attention as a promising strategy in cancer therapy [41, 42]. Given the pivotal role of USP10 in HNSCC progression, the discovery and development of specific USP10 inhibitors represent a potentially effective therapeutic approach for this malignancy. Several compounds have previously been reported to inhibit USP10 activity. For instance, Compound D1, featuring a quinoline-4(1H)-one core structure, was identified as a novel USP10 inhibitor that promotes YAP degradation and induces S-phase arrest in hepatocellular carcinoma cells [43]. Spautin-1 functions as an antagonist of both USP10 and USP13 and has shown synergistic effects with cisplatin in melanoma treatment [44]. Another compound, Wu-5, enhances crenolanib-induced apoptosis in acute myeloid leukemia (AML) cells by suppressing the FLT3 and AMPK signaling pathways [45]. However, the in vivo antitumor efficacy and target specificity of Compound D1 remain unverified [43]. Moreover, Spautin-1 lacks direct binding evidence to USP10, raising concerns about its selectivity [44]. Although Wu-5 exhibits potential against AML, its associated clinical toxicity profile is still unclear [45]. These limitations underscore the urgent need for identifying new, highly specific, and potent USP10 inhibitors for cancer treatment.

Drug repurposing—redeploying approved drugs for new therapeutic indications—has emerged as an efficient and cost-effective strategy in drug development. Compounds within the FDA-approved Drug Library offer well-documented pharmacological profiles, safety records, and bioavailability data, making them valuable candidates for rapid screening and translational application. In our study, we leveraged this library to screen for potential USP10 inhibitors and identified RAF265—a potent, orally available dual inhibitor of RAF and VEGFR2—as a promising candidate. Our experimental results demonstrate that RAF265 effectively suppresses USP10 expression in HNSCC cells and subsequently reduces SCD1 protein levels in a USP10-dependent manner. Furthermore, RAF265 inhibits SCD1-mediated lipid synthesis, impedes malignant tumor behaviors, and sensitizes HNSCC cells to ferroptosis.

Although we have demonstrated that RAF265 targets USP10 to suppress the malignant progression of HNSCC and promote ferroptosis, our article still has certain limitations. Firstly, the potential synergistic effect of combining RSL3 and RAF265 both in vitro and in vivo still requires further investigation.Secondly, the correlation between SCD1 and E2F4 in HNSCC samples needs to be further explored.

In conclusion, our study elucidates the oncogenic function of USP10 in HNSCC, showing that elevated USP10 expression enhances SCD1 stability, thereby promoting lipogenesis, tumor growth, and resistance to ferroptosis. We also demonstrate that E2F4 directly activates USP10 transcription by binding to its promoter region in HNSCC. Collectively, these findings highlight the critical role of the E2F4/USP10/SCD1 signaling axis in modulating ferroptotic cell death and driving HNSCC progression, suggesting that targeting this pathway—particularly with repurposed agents like RAF265—may offer a novel therapeutic avenue for HNSCC management.

## Material and method

### Reagents

The antibodies used in this study: Ki67 (Abcam, ab15580,1:500), Caspase-3 (Abcam, ab32351, 1:100), E2F4 (CST, 40291S, 1: 1000), Actin (1:1000, sc-1616, Santa Cruz Biotechnology), USP10 (Proteintech, 19374-1-AP,1:5000), SCD1(Abcam,ab236868,1:1000), Ub (1:1000, SC-47721, Santa Cruz Biotechnology), RSL3 was purchased from MCE (HY-100218A), Ferr-1 were purchased from Sigma-Aldrich (SML0583), Z-VAD-FMK (S7023), 3-MA (S2767) were purchased from Selleck.

### Cell culture, gene knockdown and knockout

HNSCC cell Lines UM-SCC-1 were cultured in RPMI 1640. CAL-27 and UM-SCC6 cells were cultured in DMEM medium. To generate USP10, E2F4 or SCD1-depleting HNSCC cells, we inserted shRNA sequences of USP10, E2F4 and SCD1 into the pLKO.1-puro vector. The shRNA sequences were listed: human USP10: 5- CCCATGATAGACAGCTTTGTT-3; E2F4#1: 5-CCCTCTCTTCATTTCGGCTTT-3 and E2F4#2: 5-GACCTCTTTGATGTGCCTGTT-3; SCD1:5-CTTGGATTTGATGTCGTCTTT-3. We transfected these plasmids together with pPAX2 and pMD2 lentiviral packaging systems into 293 T cells and obtained the lentivirus. Then, we used the lentivirus to infect HNSCC cells. The stable cells were selected with 5μg/mL puromycin for 2 days. To generate USP10 knockout HNSCC cells, sgRNA was constructed into the lentiCRISPRv2 vector and packaged in lentivirus, and infected cells were screened with puromycin to obtain the stable cell line. The sgRNA sequence was listed: 5-CACCgGCCTGGGTACTGGCAGTCGA-3.

### Quantitative reverse transcription PCR (qRT‒PCR)

Total RNA of UM-SCC-1 and UM-SCC6 with or without E2F4 knockdown or overexpression was isolated using TRIzol (Invitrogen). Total RNAs isolated from the indicated cells were synthesized into cDNA using the PrimeScriptTM RT reagent kit (Takara, RR047A). The expression levels of USP10 were normalized to those of β-actin. Changes in gene expression were determined using the 2^−ΔΔCT^ method. The primers for USP10. Forward:5-GATCAATAAAGGGAACTGGTGC-3; Reverse:5- AGCTGTCTATCATGGGTGTTG-3. Actin, Forward: 5-CACCTTCTACAATGAGCTGCGTGTG-3; Reverse: 5-ATAGCACAGCCTGGATAGCAACGTAC-3.

### Western blot

Total protein was extracted from HNSCC cells, followed by denaturation and separation via SDS-polyacrylamide gel electrophoresis. Proteins were then transferred onto nitrocellulose membranes. Membranes were blocked with 5% non-fat milk for 2 h at room temperature and subsequently incubated overnight at 4 °C with primary antibodies. The Full and uncropped western blots are shown in the Supplementary material.

### Colony formation and migration assays

For the colony formation assay, the indicated HNSCC cells were seeded into a 6-well plate and cultured at 37 °C in a 5% CO_2_ incubator for one week. The indicated cell colonies were fixed with methanol for 15 min and stained with 0.1% crystal violet for 15 min. Images of cell colonies were captured using the Bio-Rad ChemiDoc XRS+ system and quantified with the ImageJ program.

The indicated HNSCC cells were seeded in a 24-well Transwell plate with 8-mm polyethylene terephthalate membrane filters (Corning, 3422). After incubation at 37 °C with 5% CO_2_, the cells that passed through the membrane were fixed with 4% formaldehyde for 30 min and stained with 0.1% crystal violet for 20 min. After wiping off the upper layer of nonmigrated or noninvasive cells with a cotton swab, cells were counted by light microscopy.

### Lipid peroxidation assay and lipidomics

The indicated HNSCC Cells were seeded in 6-well plates and treated with RSL3. Then treated with 5 μM BODIPY 581/591 C11 (D3861, Thermo Fisher Scientific) and incubated at 37 °C for 30 min. The cells were then washed twice with 1 mL 1× PBS, digested with trypsin to obtain cell pellets, and suspended in 500 μL PBS. A BD Accuri C6 Plus personal flow cytometer was used to analyze changes in fluorescence.

The indicated UM-SCC1 cells were collected, and untargeted lipidomics analysis was performed using ultra-high-performance liquid chromatography–tandem mass spectrometry (UHPLC-MS).

### Chromatin immunoprecipitation (ChIP) assay

UM-SCC1 cells with or without E2F4 overexpression were crosslinked with 1% formaldehyde for 10 min at room temperature. The ChIP assay was performed according to the manufacturer’s instructions using the anti-E2F4 antibody and a kit (EZ-ChIP, 17-371 Millipore, Merck KGaA, Darmstadt, Germany). Anti-rabbit IgG was used as the control. The bound DNA fragments were eluted and amplified by qRT-PCR.

### Promoter reporters and dual-luciferase assay

The promoter of USP10 and the matching mutant were constructed into a pGL3-basic vector. Luciferase activity was measured in a 1.5-ml Eppendorf tube with a Promega Dual-Luciferases Reporter Assay kit. Relative Renilla luciferase activity was normalized to firefly luciferase activity.

### In vivo tumor formation and metastasis assays

All the mice were randomly divided into the indicated groups. 5 × 10^6^ USP10 depleting CAL-27 cells or the control cells were injected subcutaneously into female nude mice. Tumor size was measured at regular intervals of 2 days, beginning at 25 days after injection. The animals were executed at 43 days post-injection. The tumor weight and volume were counted. 1 × 10^7^ the indicated UM-SCC1 cells/mouse was injected subcutaneously into NOD SCID female mice. Tumor size was measured at regular intervals of 2 days, beginning at 13 days after injection. The animals were executed at 28 days post-injection. The tumor weight and volume were counted.

### Light microscopy and TEM

For light microscopy, the indicated cells were cultured in 6-well plates and treated with reagents as indicated. Phase-contrast images were captured using a Leica microscope equipped with a 10× phase-contrast objective.

For ultrastructural analysis of mitochondria, TEM was used. The indicated HNSCC cells were treated with or without RSL3, and fixed with 2.5% glutaraldehydeat 4°C for 2.5 h, washed 3 times with 0.1 M PBS, and postfixed in 1% OsO4 for 2 h at 4°C. The samples were then dehydrated through an ethanol gradient and subsequently embedded in Spurr’s resin. Ultrathin sections were then collected and stained with either uranyl acetate or lead citrate and examined using a JEOL 1200EX transmission electron microscope.

### Immunohistochemistry and lung histology

The HNSCC tissue microarrays purchased from Bioaitech Company(Xi’an, China) contain 45cancer tumors together and 9 normal tissues. We performed immunohistochemical (IHC) staining for USP10 and SCD1 on the same paraffin-embedded tissue blocks that were used for clinical diagnosis.

### Statistical analyses

Statistically significant difference was investigated using GraphPad Prism 8. Comparisons of two groups were analyzed utilizing Student’s *t* test. The correlation analysis was determined by Spearman’s correlation. *P* < 0.05 was defined as a significant difference.

### Ethics statements

All procedures followed were in accordance with the 1996 National Institute of Health Guide for the Care and Use of Laboratory Animals and the ethical standards approved by the Institutional Animal Care and Use Committee of Dalian Medical University (Approved number XL250623086).

## Supplementary information

Supplementary Figures and Figure Legends

Uncropped wb

## Data Availability

The datasets used or analyzed during the current study are available from the corresponding author on reasonable request.
