# Supplementary material for: Targeting USP10/SCD1 axis by RAF265 suppresses lipogenesis and induced ferroptosis in head and neck squamous cell carcinoma
Source: Cell Death Discov. 2026 Jun 2;12:325. doi: 10.1038/s41420-026-03180-1 (PMC13444109; doi:10.1038/s41420-026-03180-1)
Supplement: Supplementary file 1 [file 41420_2026_3180_MOESM2_ESM.pdf]

Figure1

Figure 1D

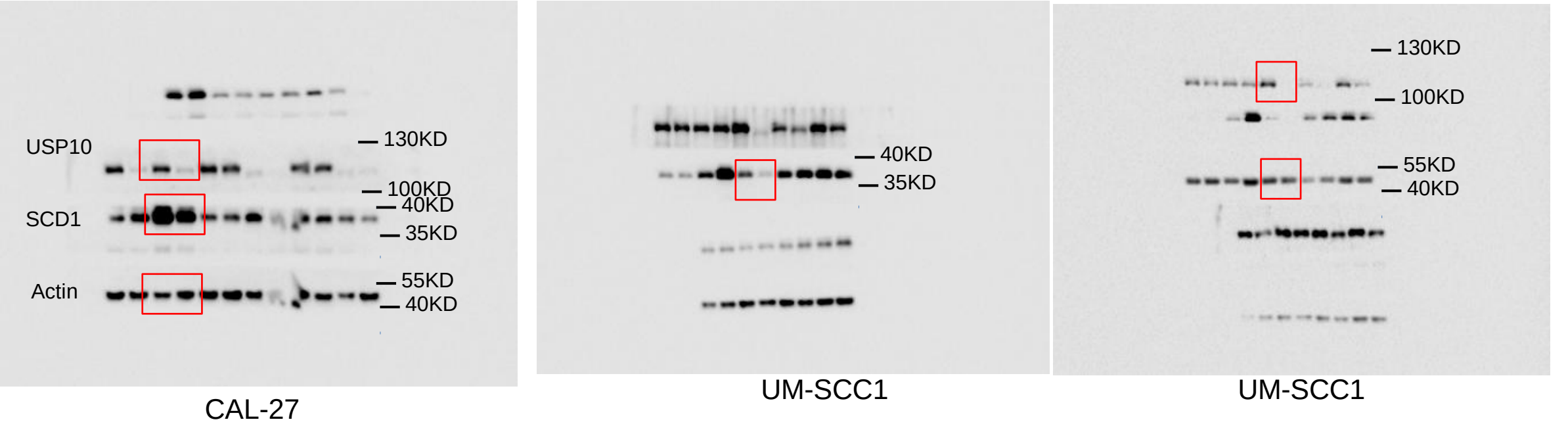

Figure 1E

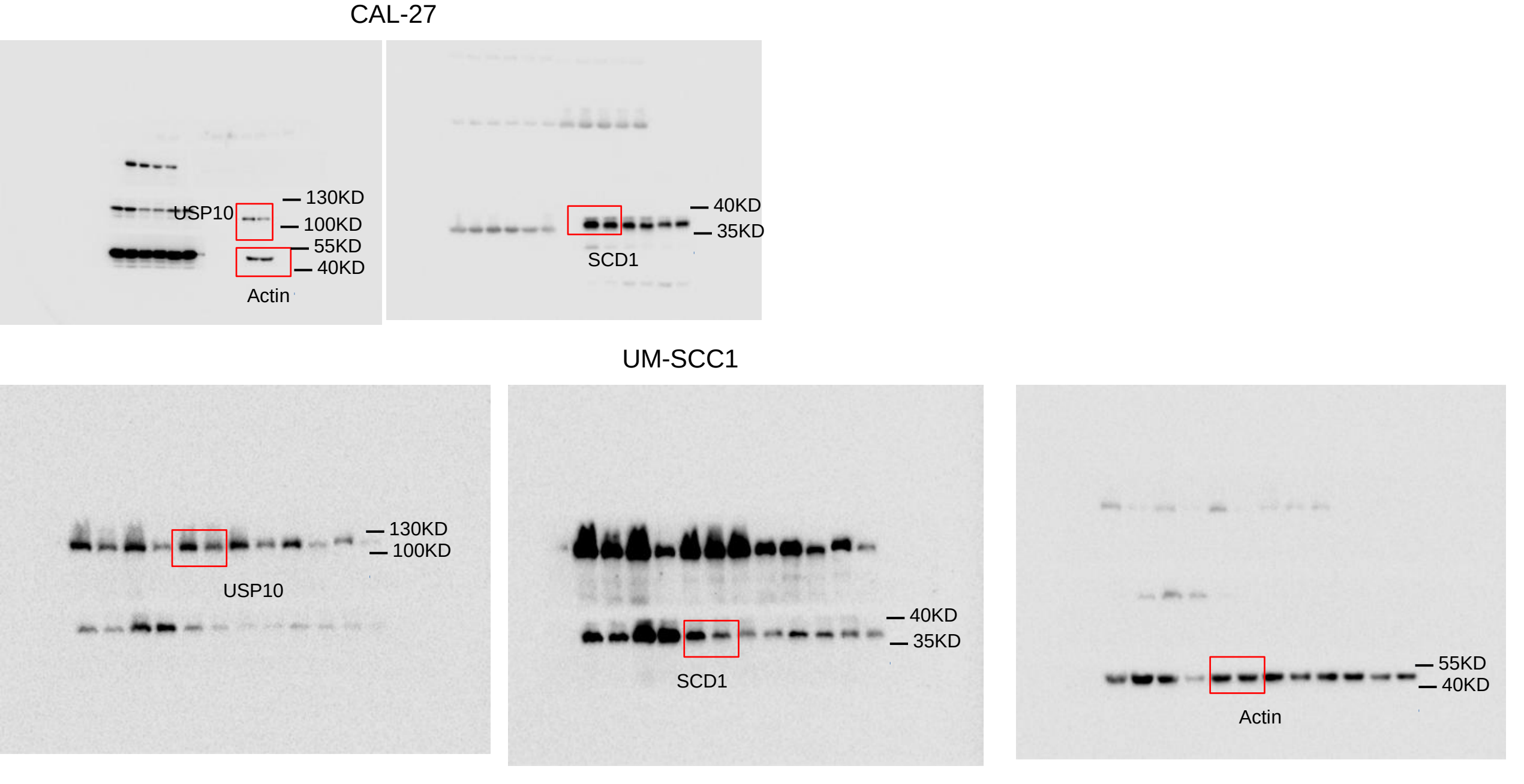

Figure 1F

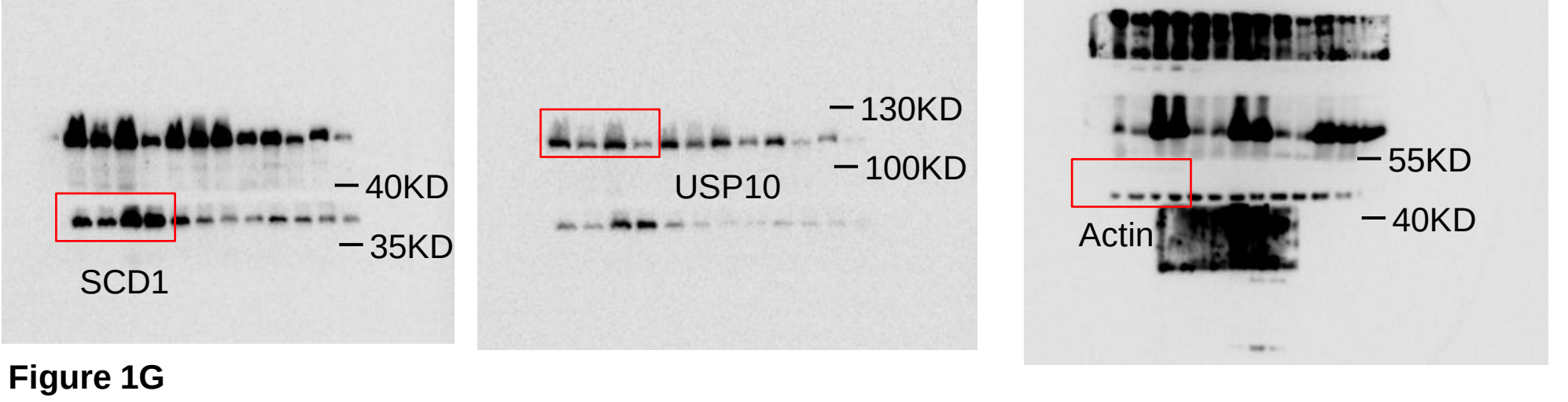

Figure 1G

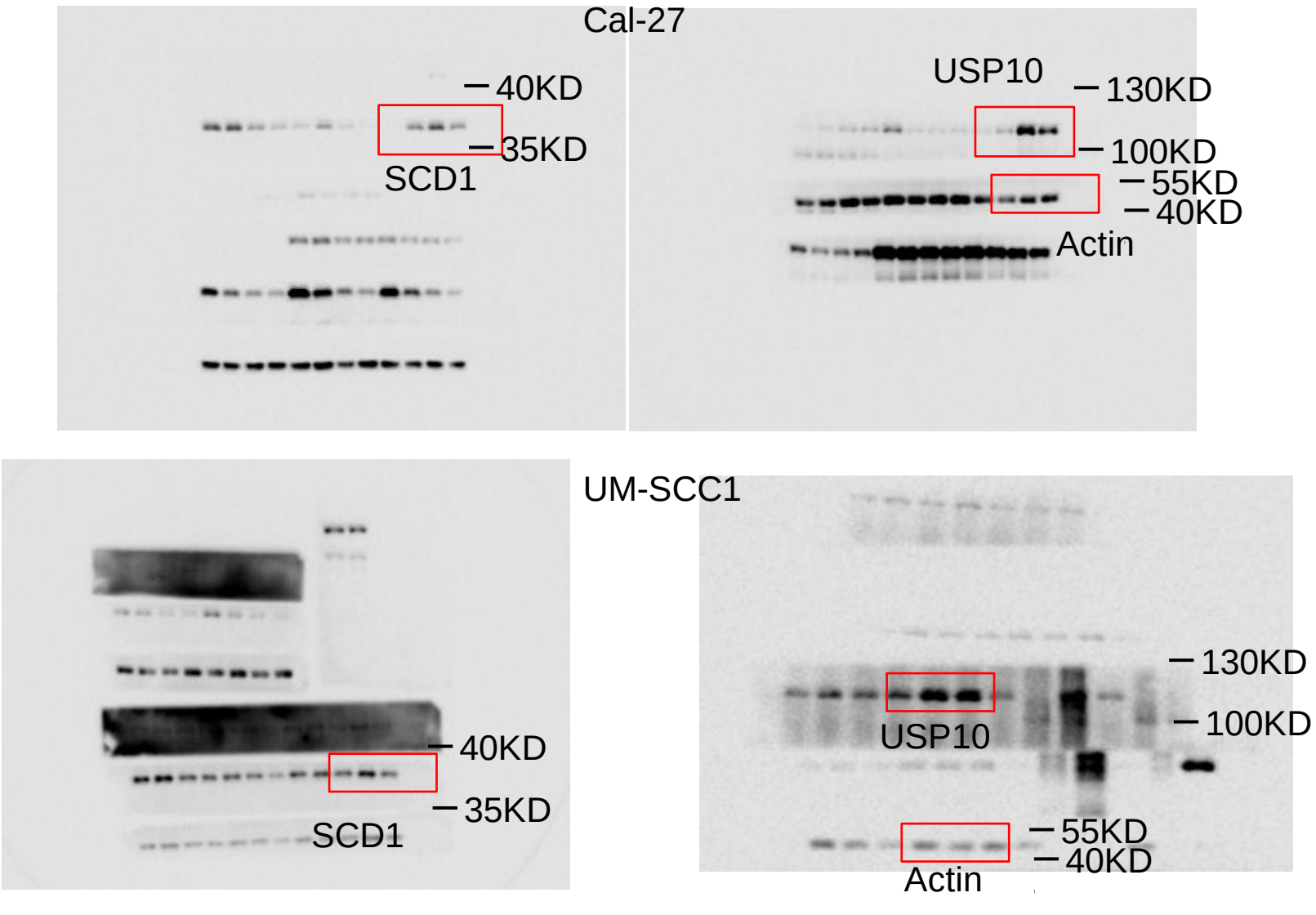

Figure 1

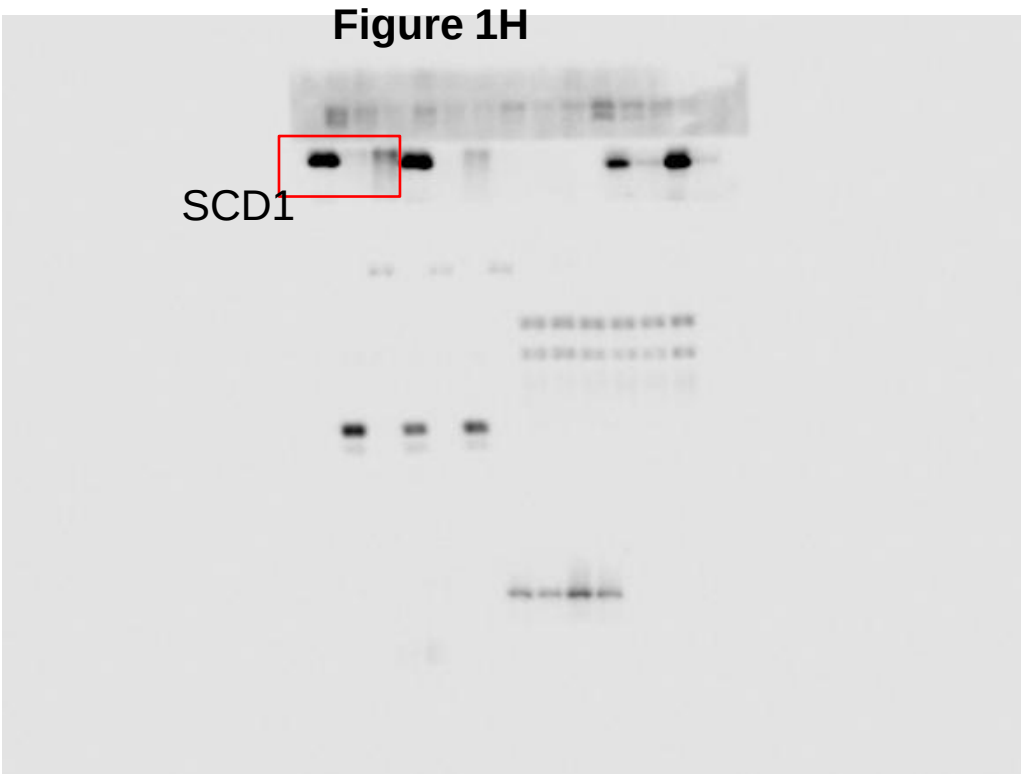
Figure 1H
SCD1

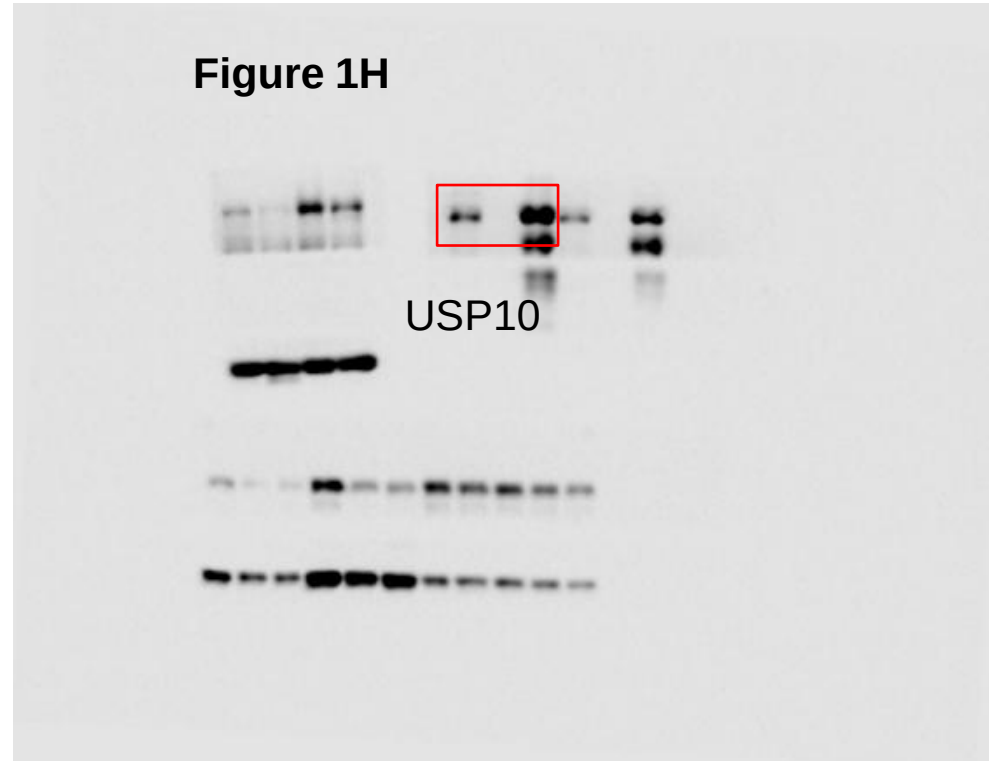
Figure 1H
USP10

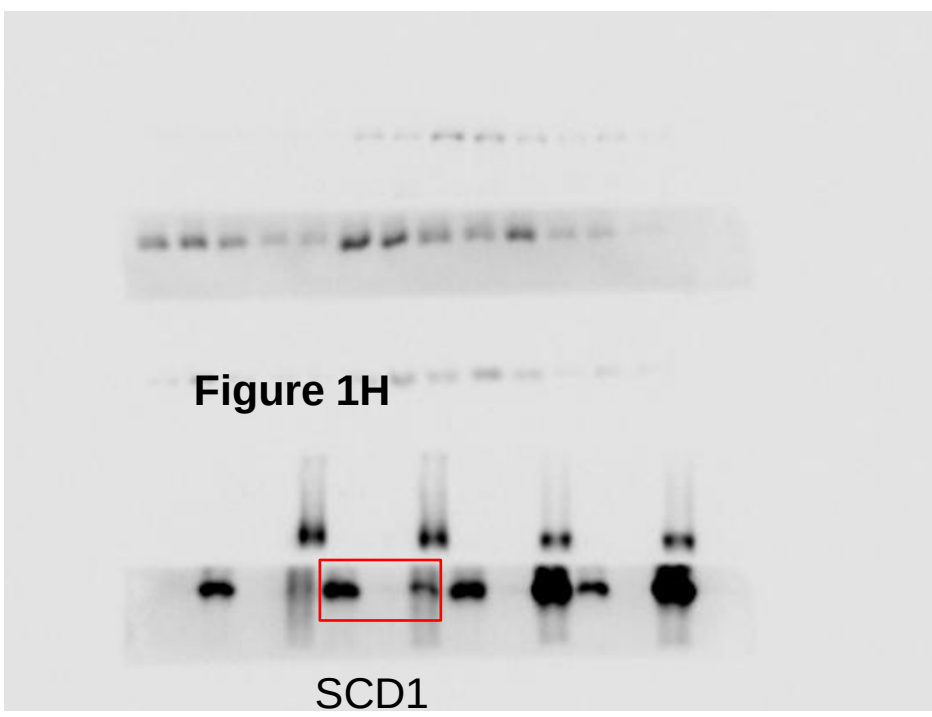
Figure 1H
SCD1

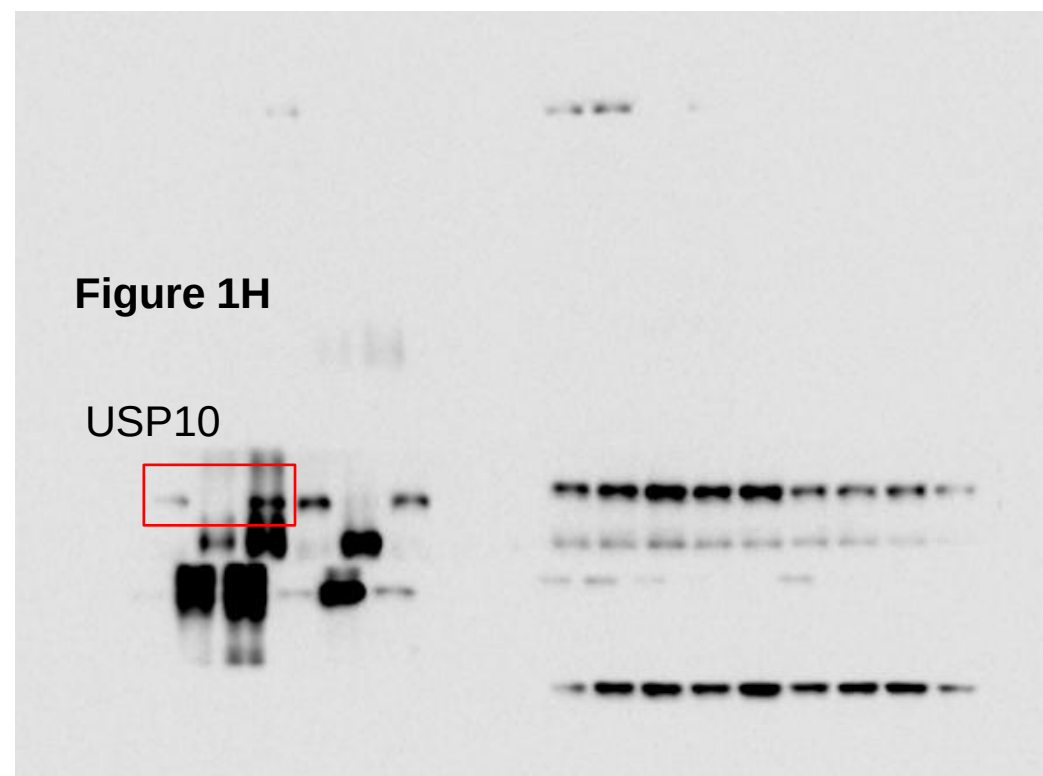
Figure 1H
USP10

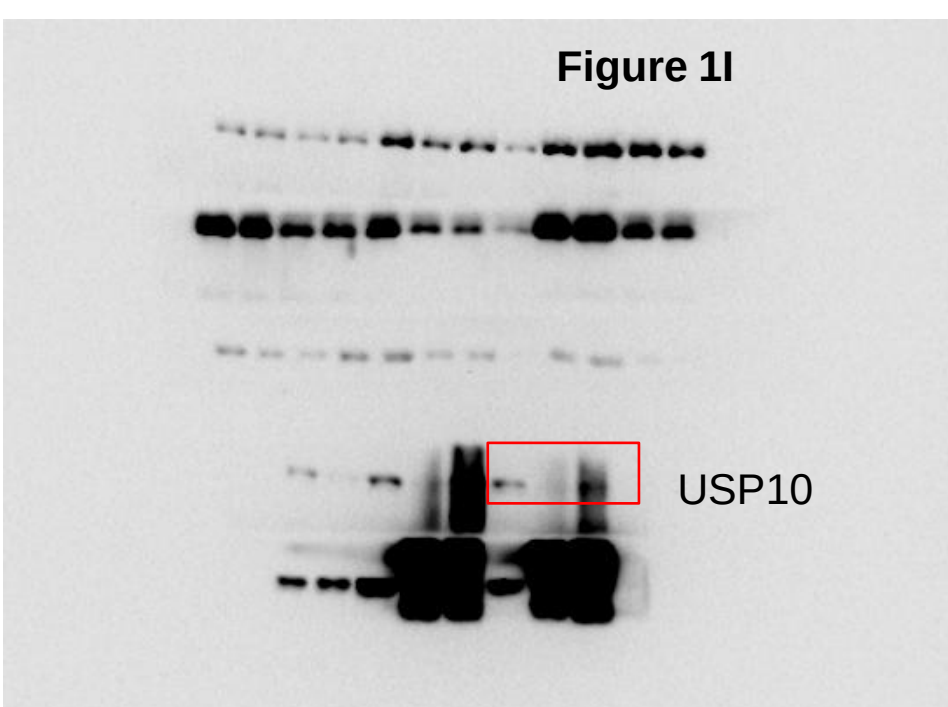
Figure 1I
USP10

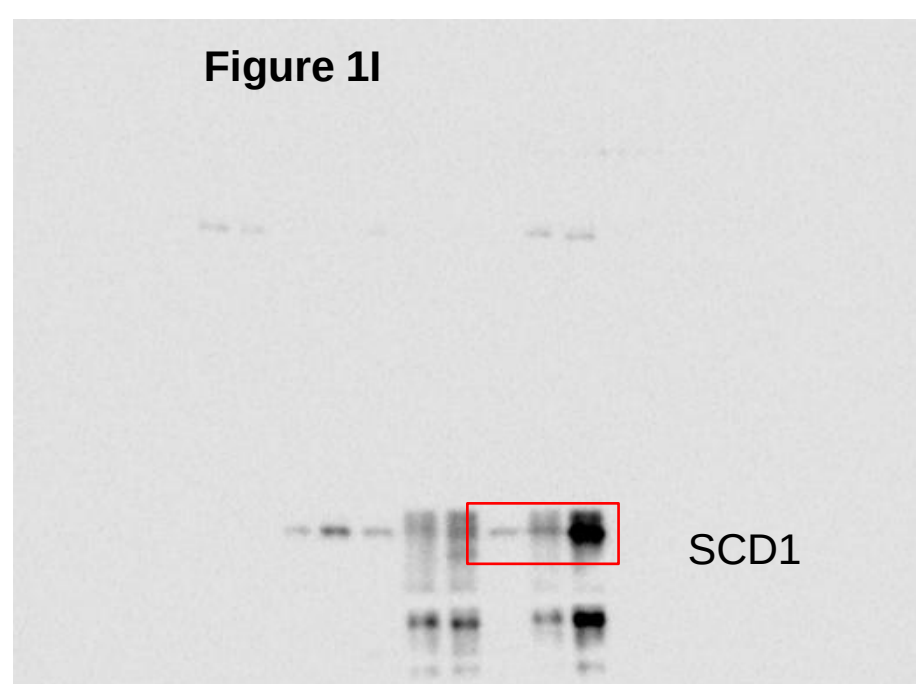
Figure 1I
SCD1

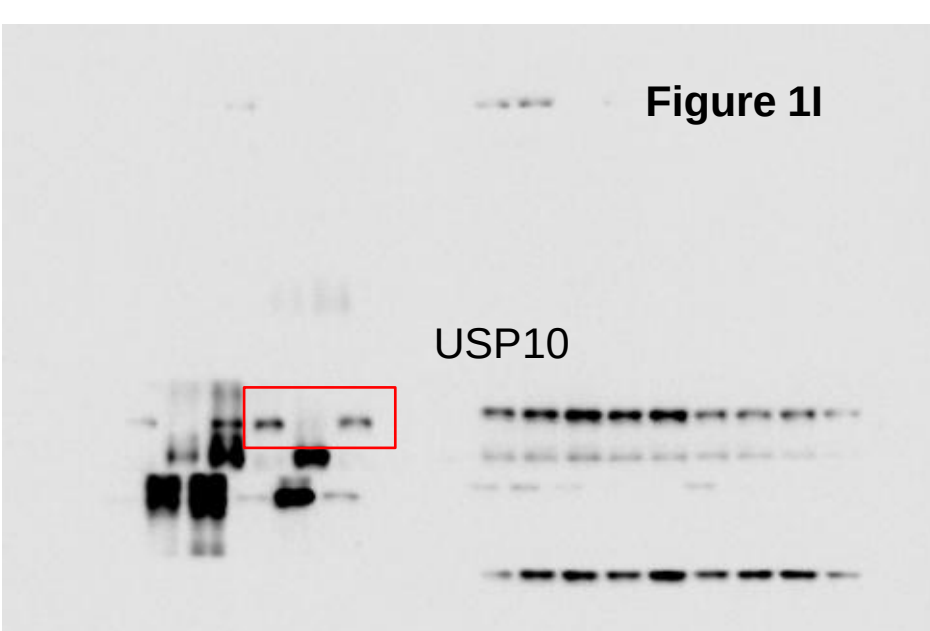
Figure 1I
USP10

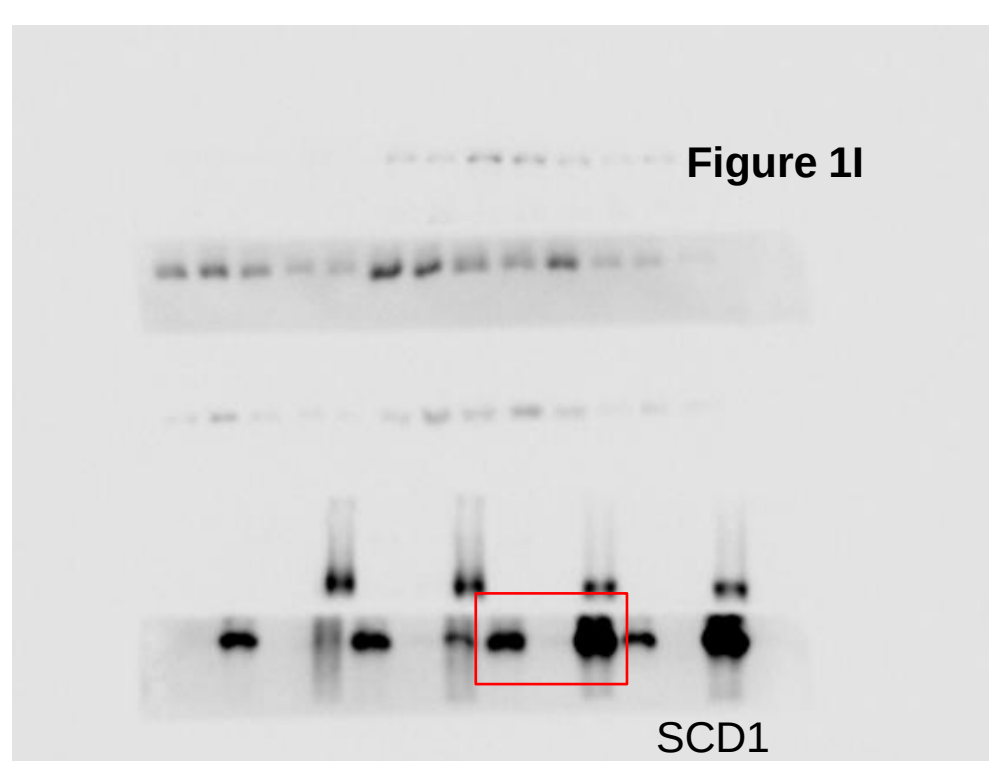
Figure 1I
SCD1

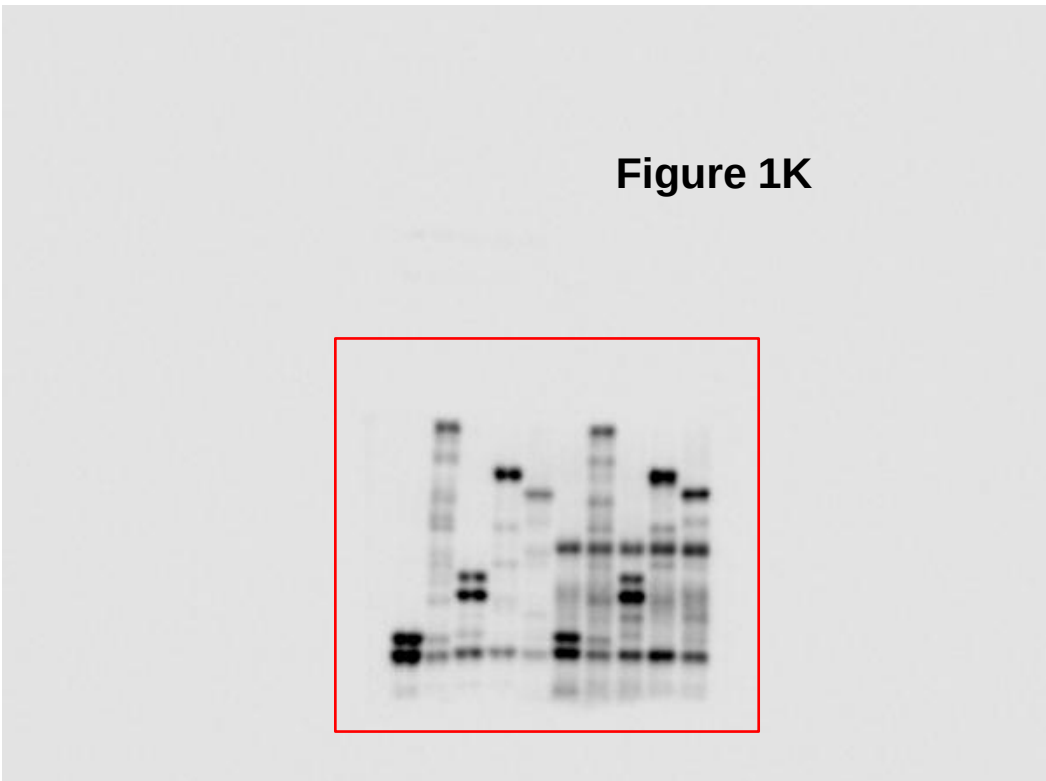
Figure 1K

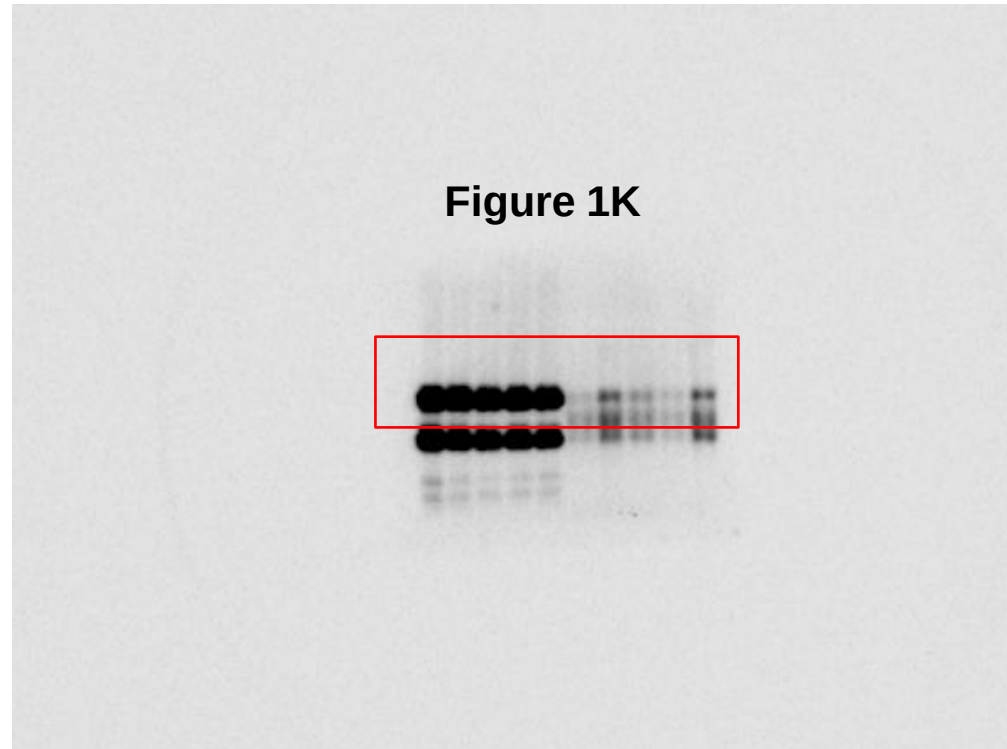
Figure 1K

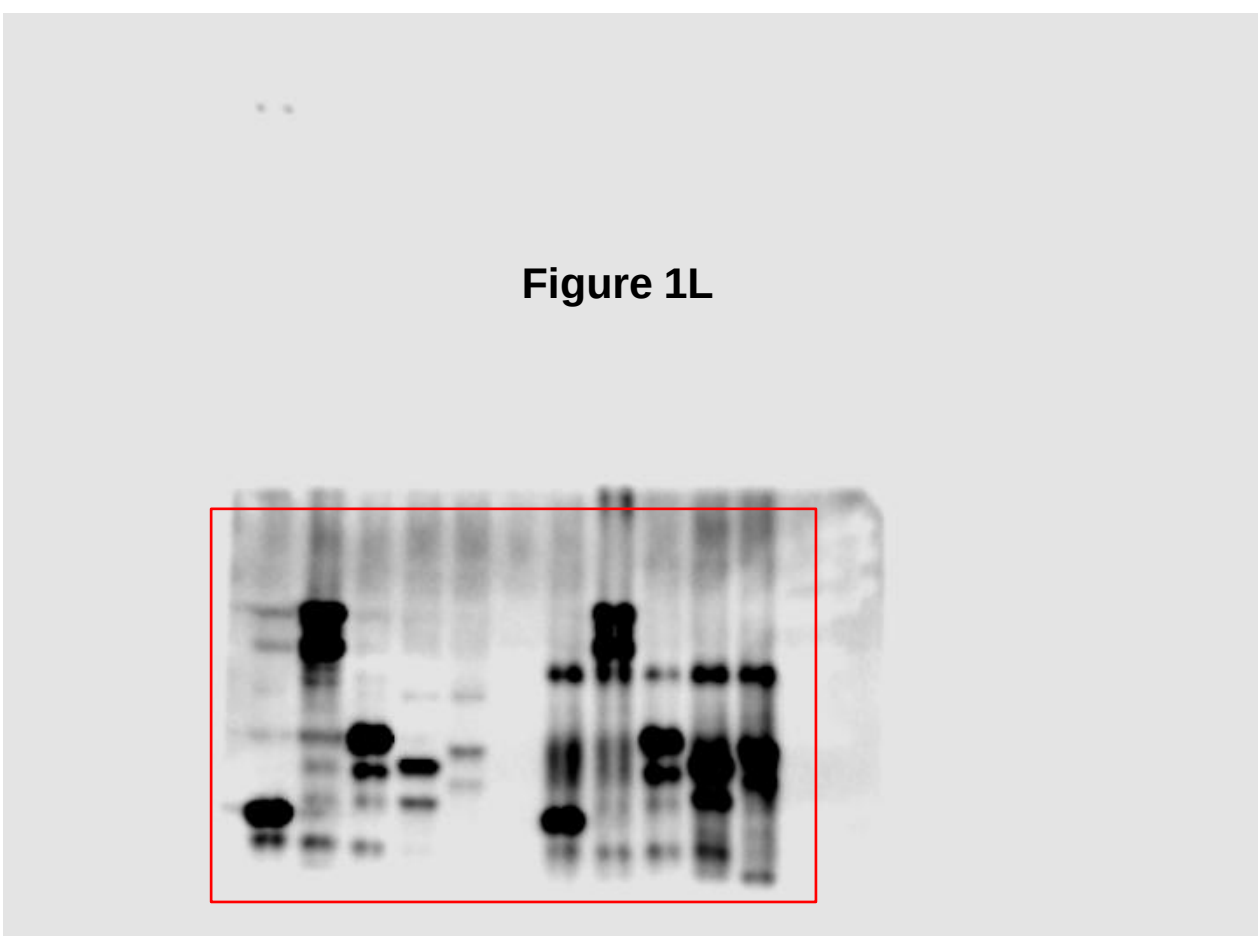
Figure 1L

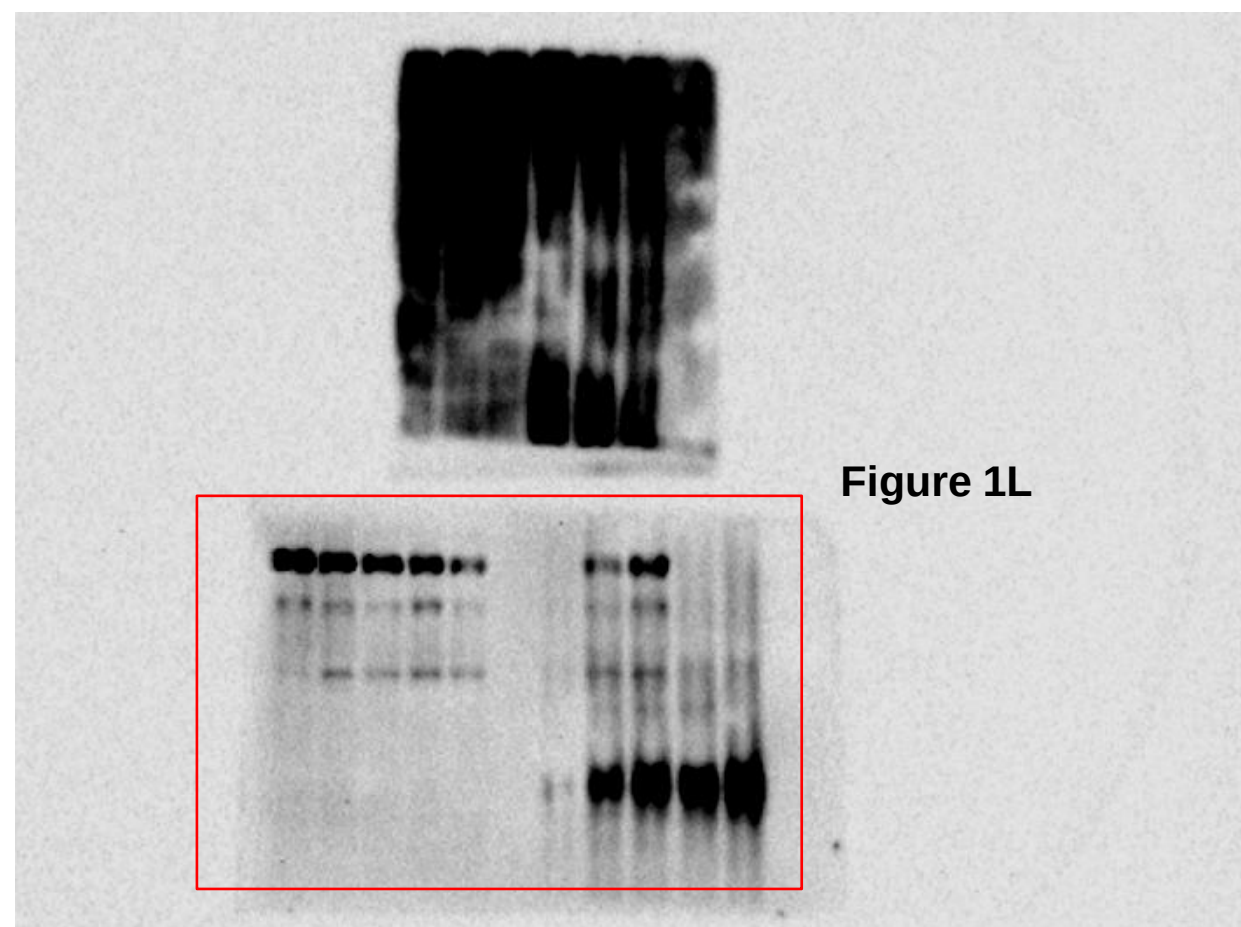
Figure 1L

Figure 2

Figure 2A

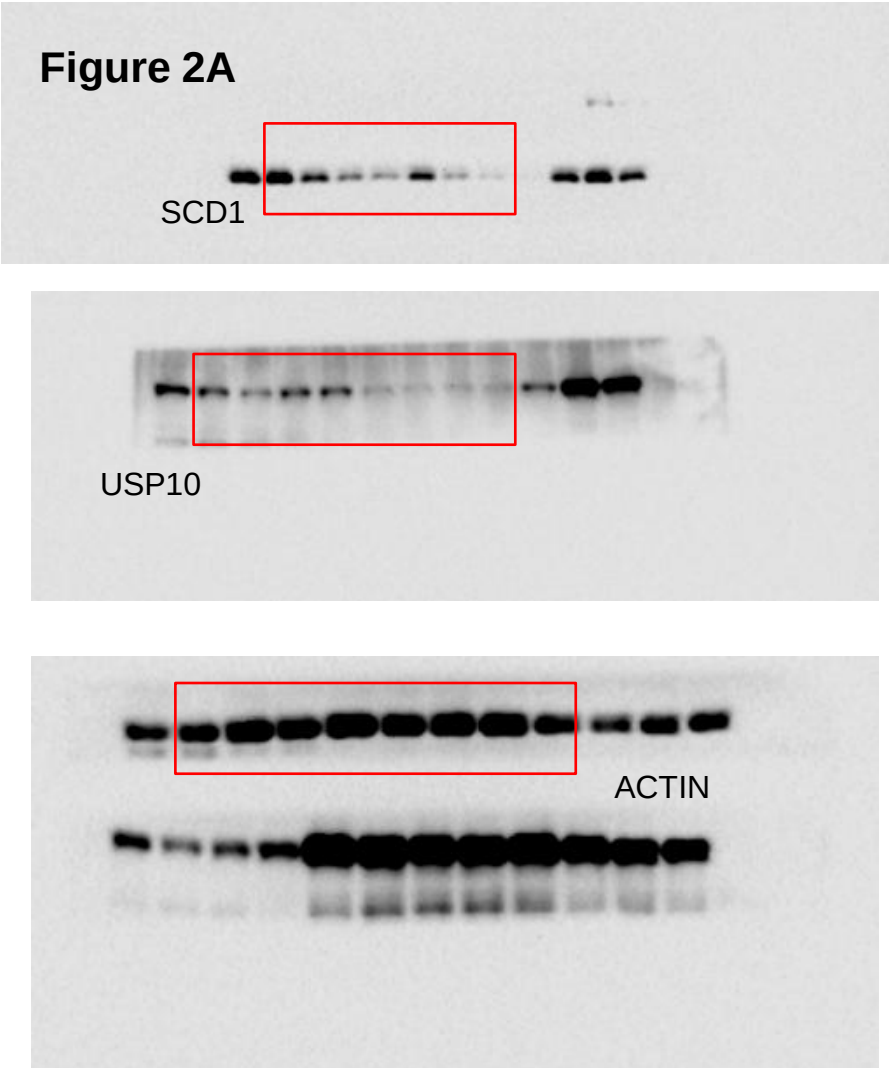

Figure 2C

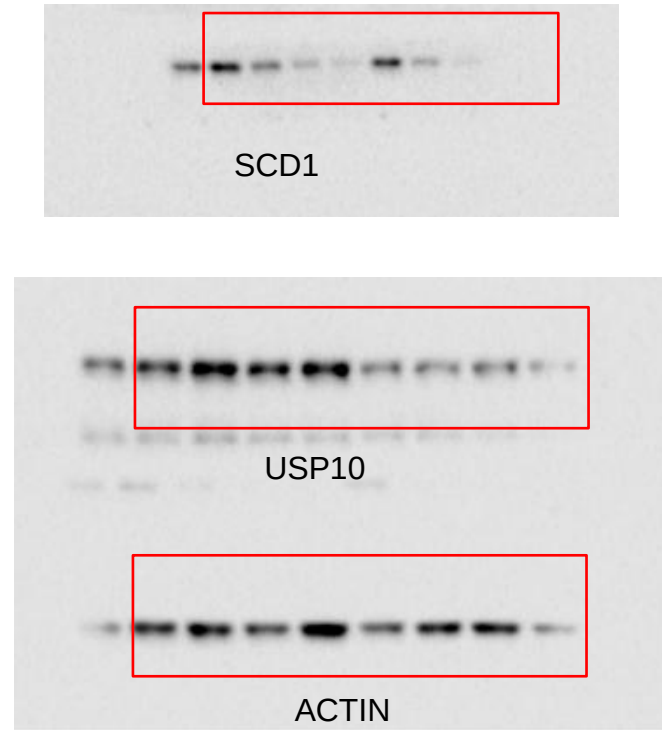

Figure 2E

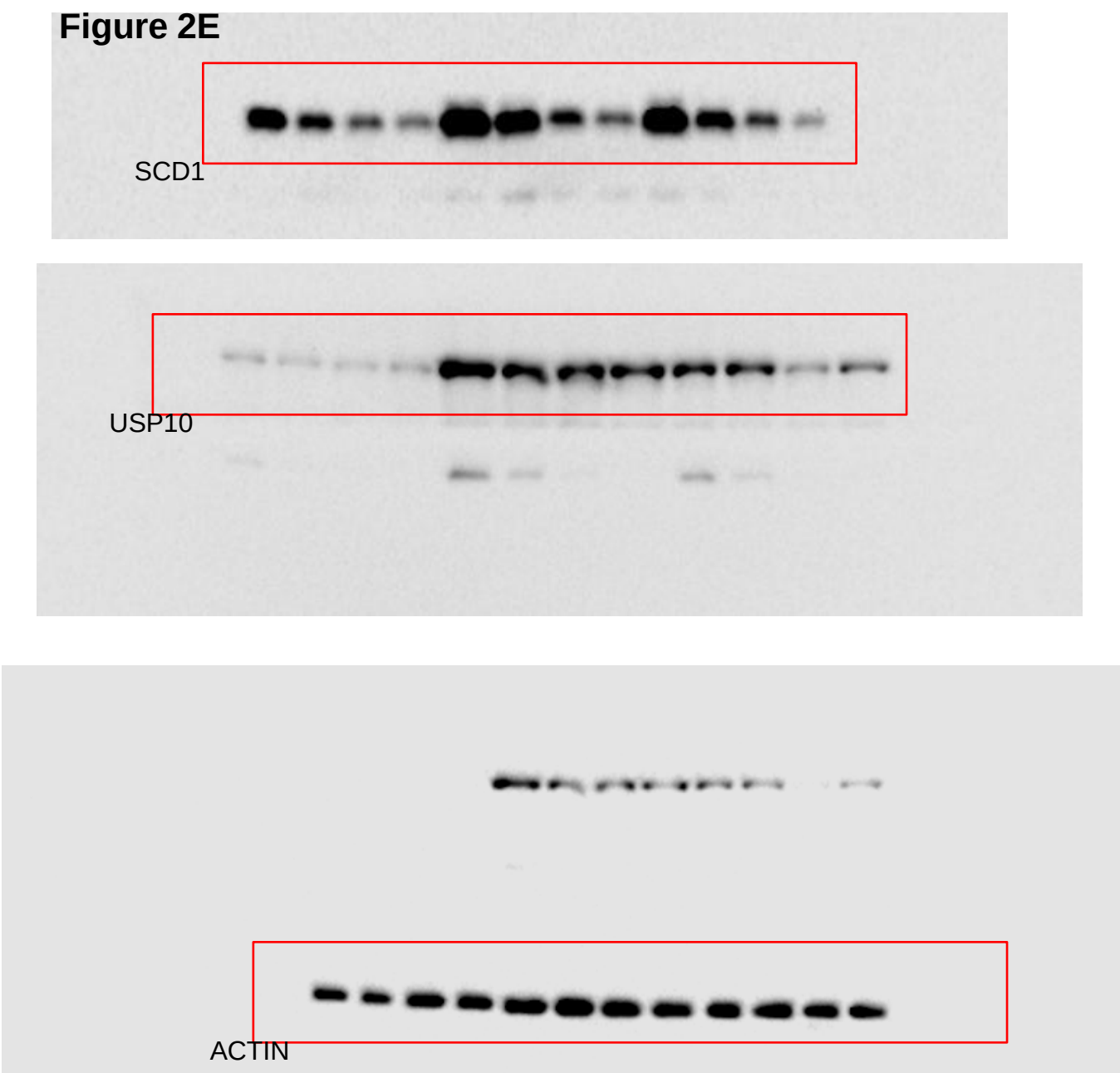

Figure 2

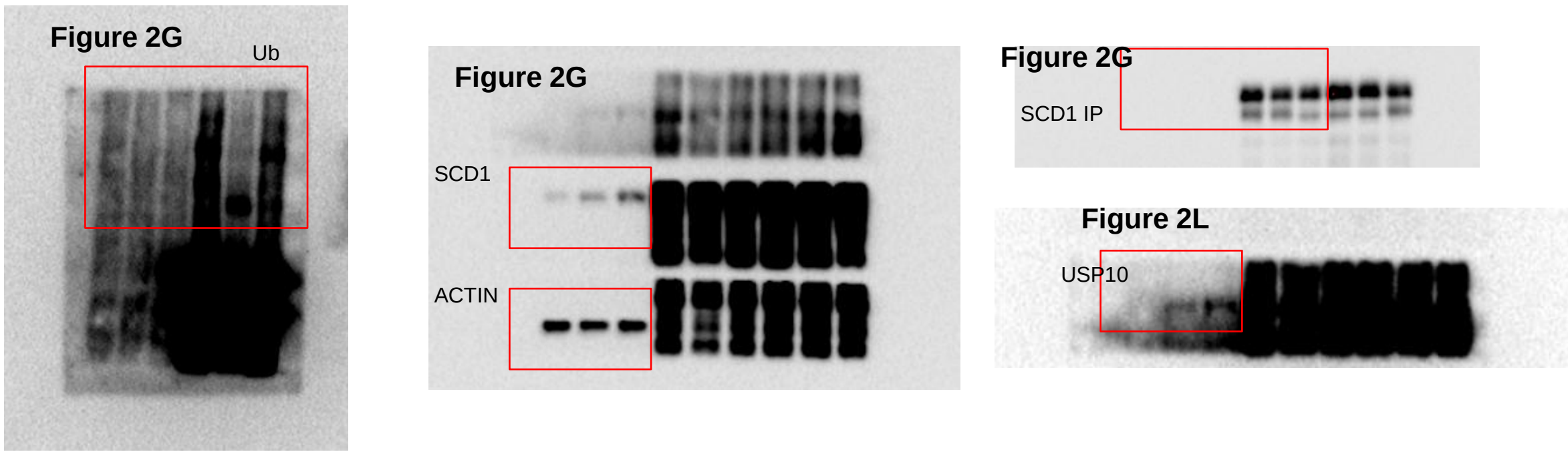

Figure 2H

Figure 2H

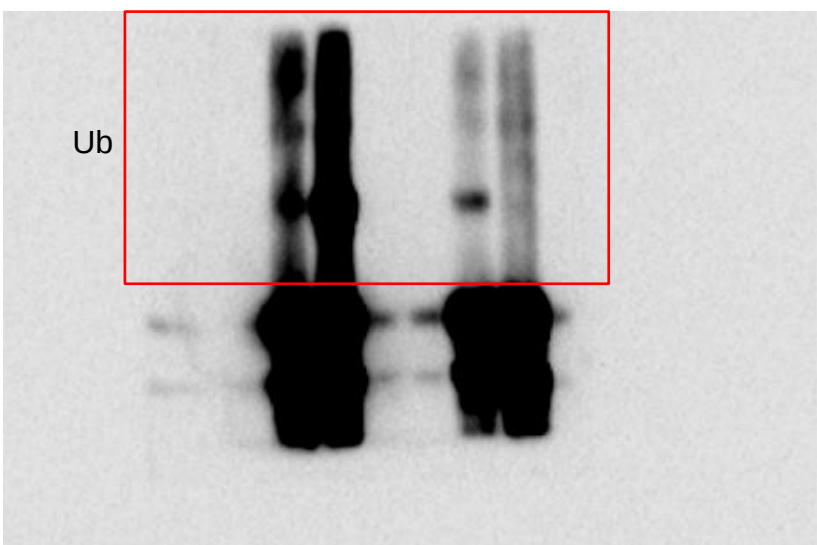

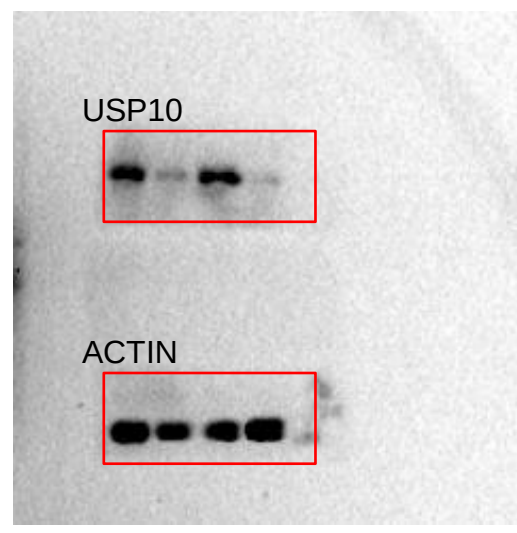

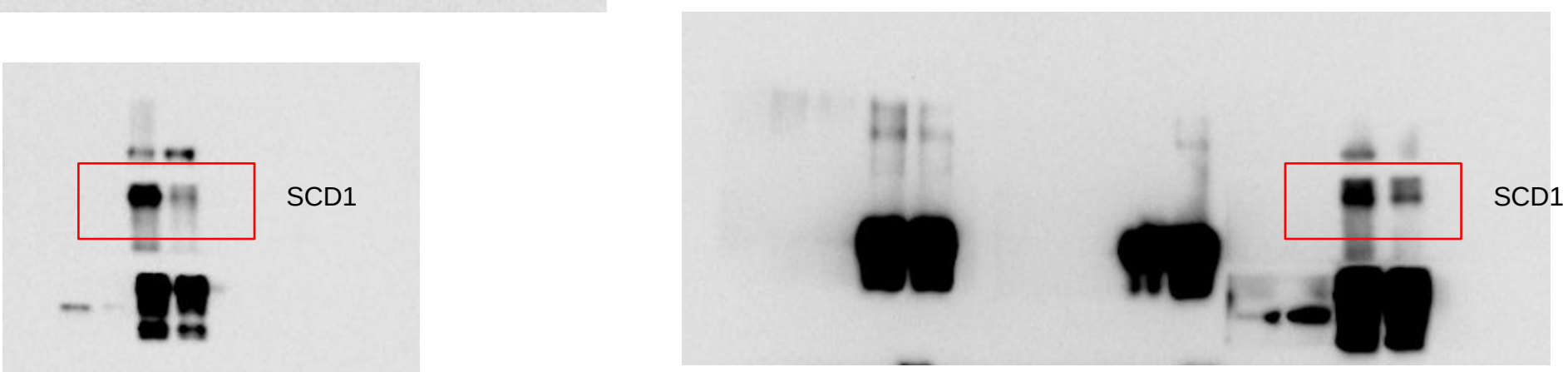

Figure 2I

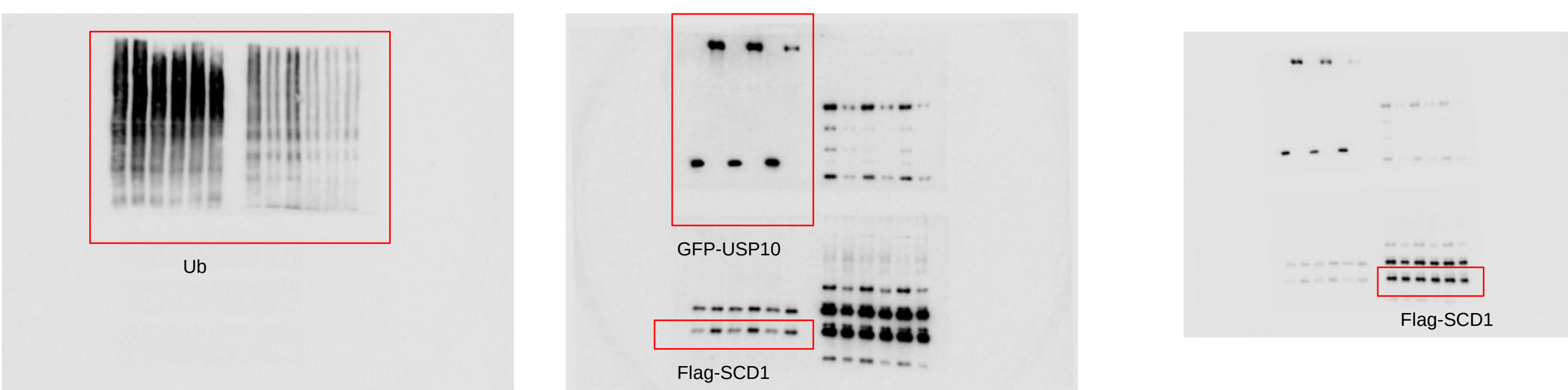

Figure 5

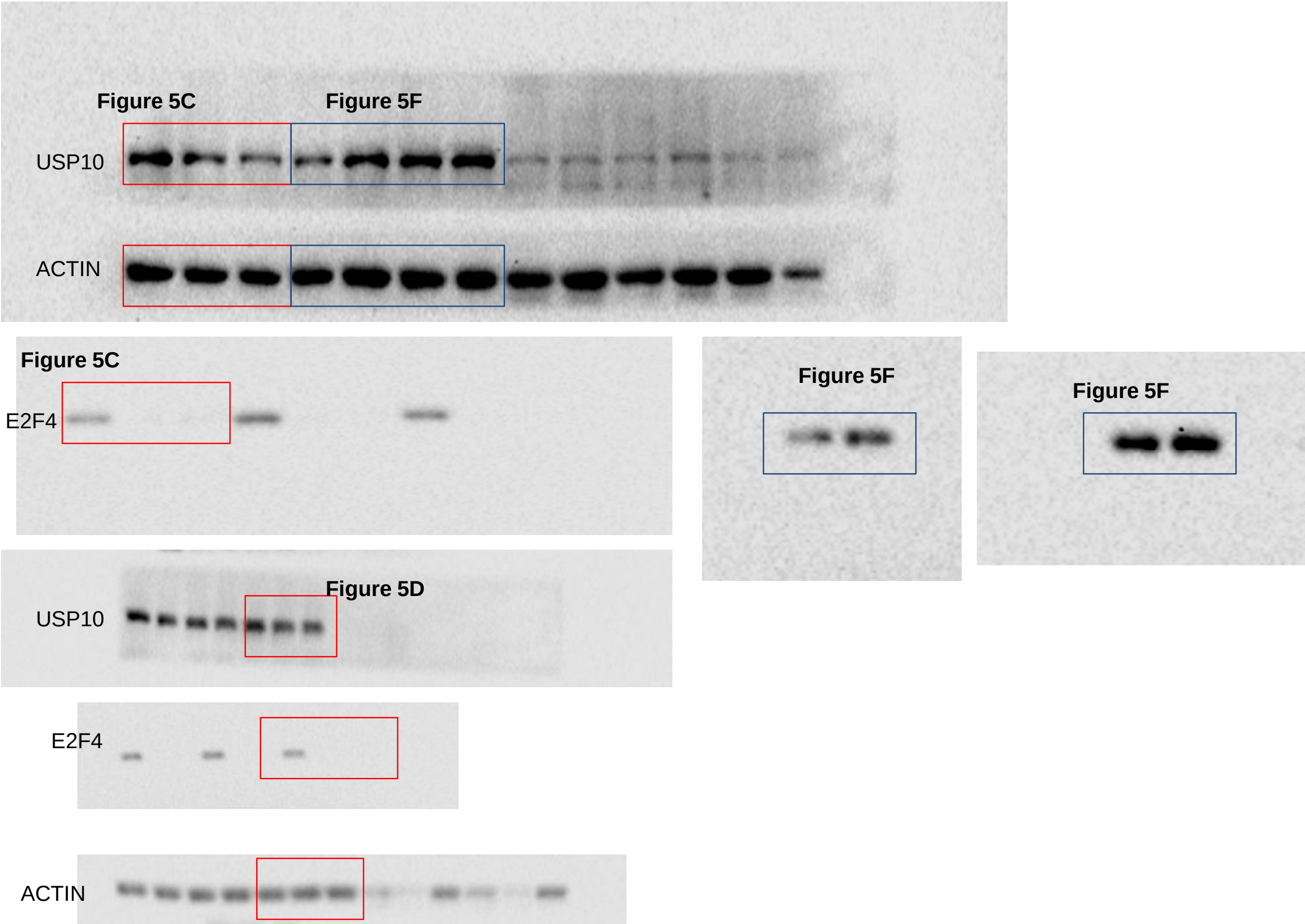
Figure 5C
Figure 5F
USP10
ACTIN
Figure 5C
E2F4
Figure 5F
Figure 5F
Figure 5D
USP10
E2F4
ACTIN

Figure 6

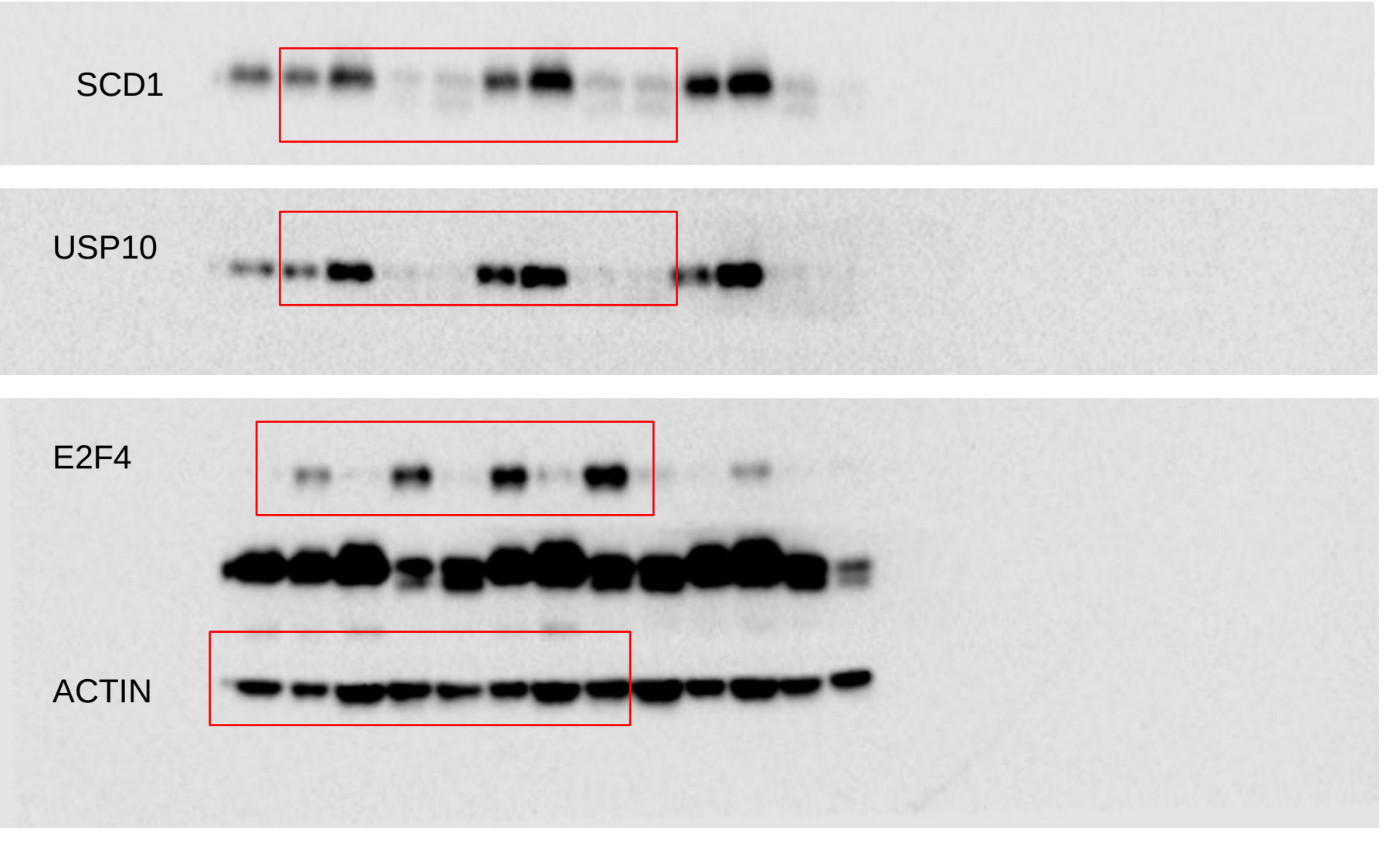

Figure 7

Figure 7D

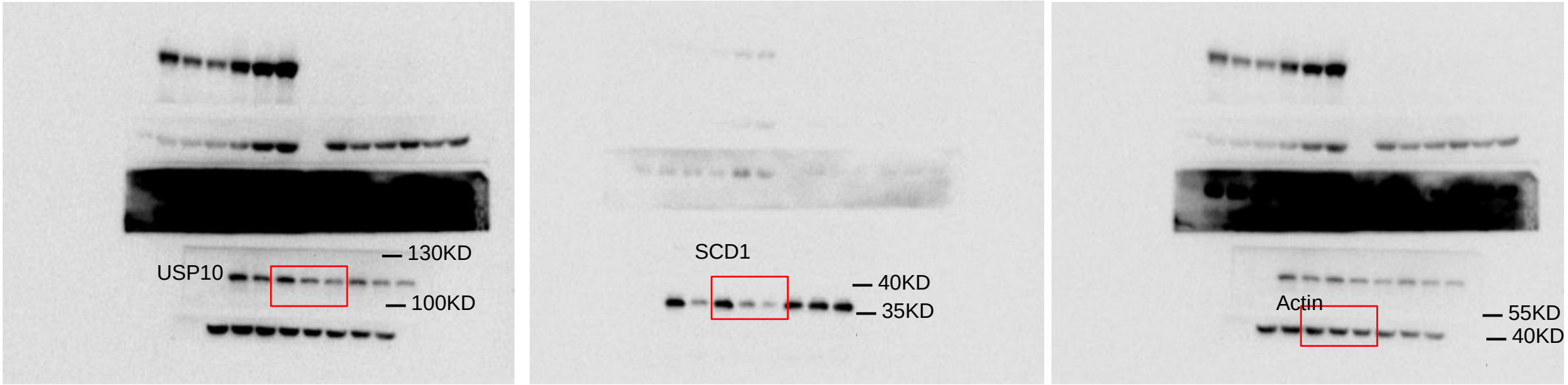

UM-SCC1

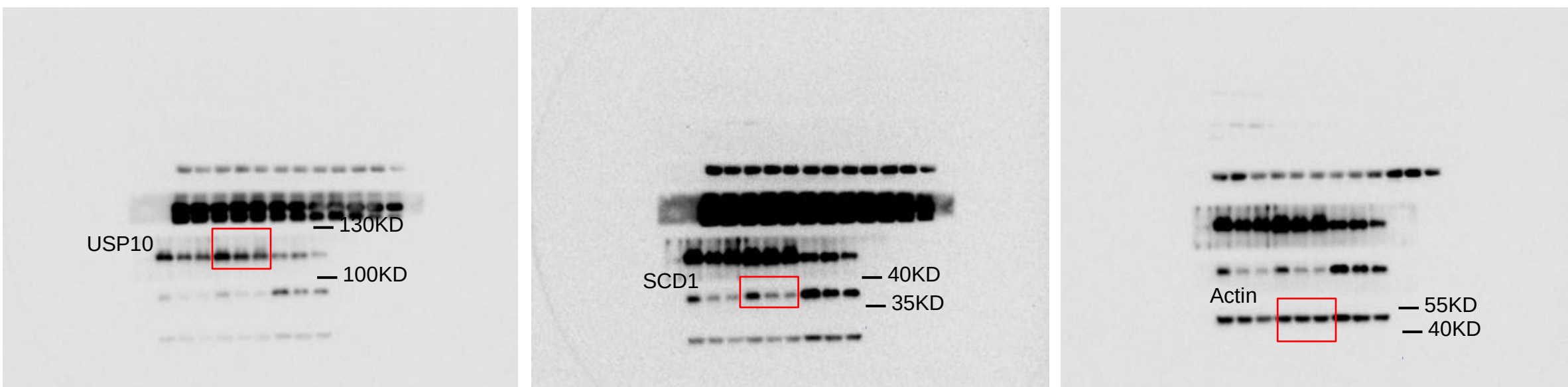

UM-SCC6

Figure 7E

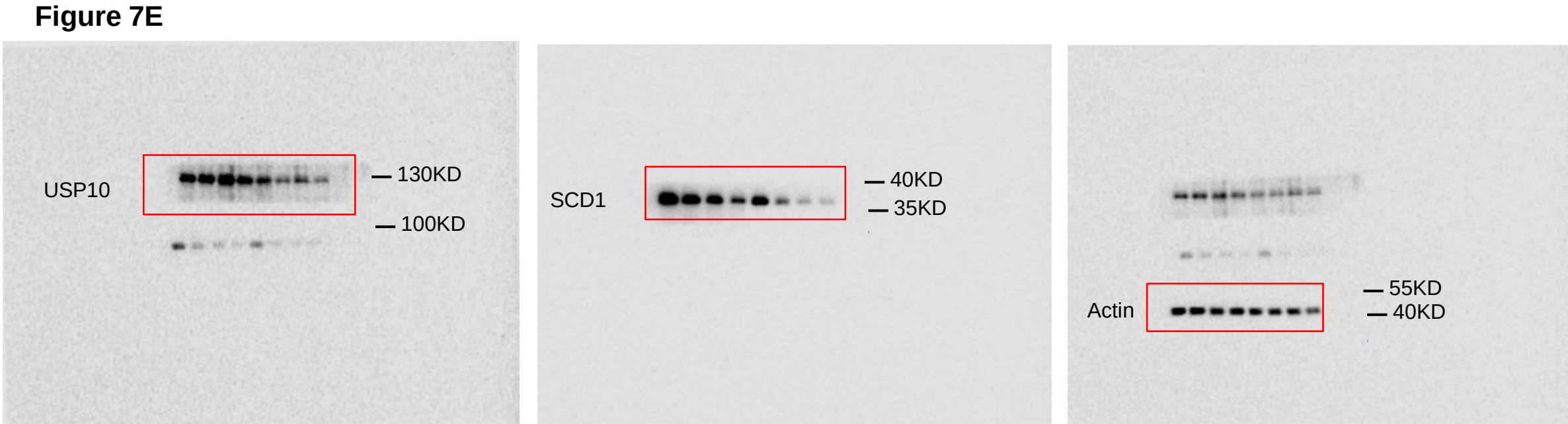

Figure 7G

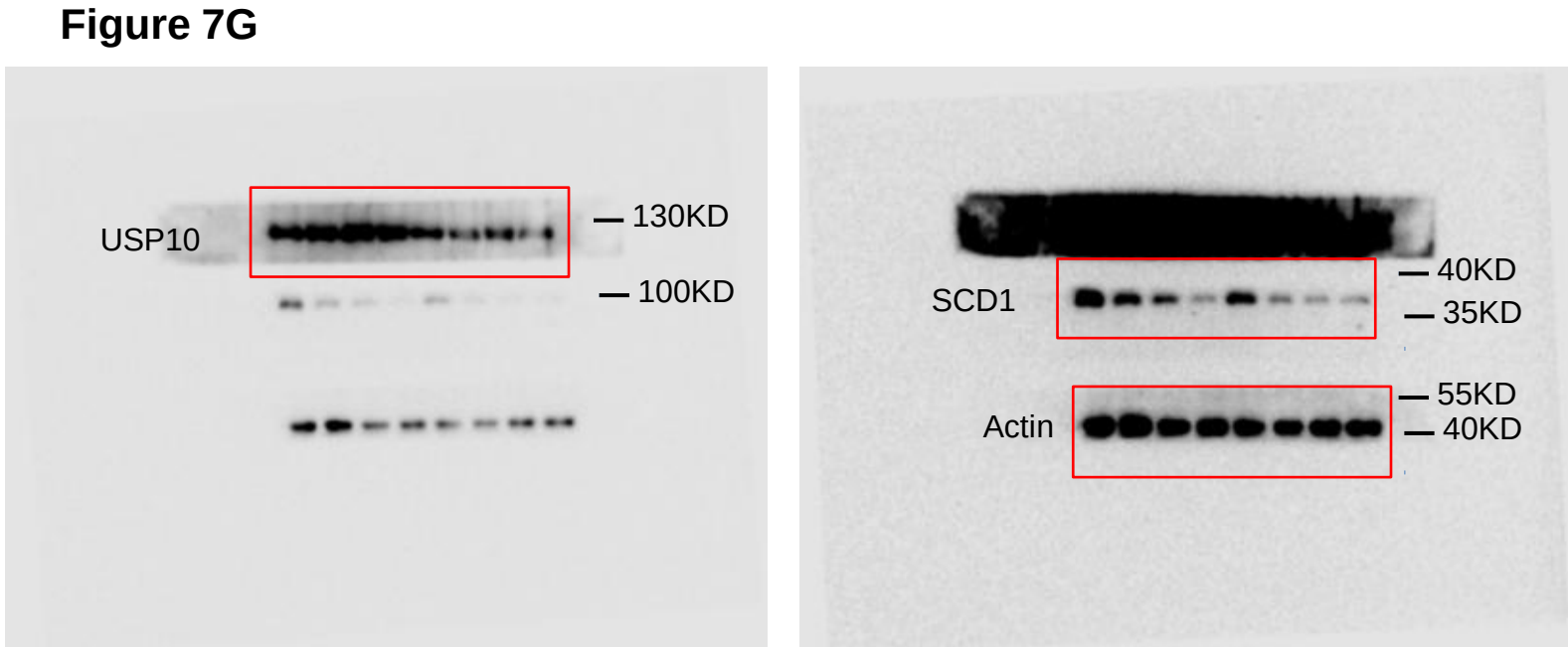

Figure 7

Figure 7I

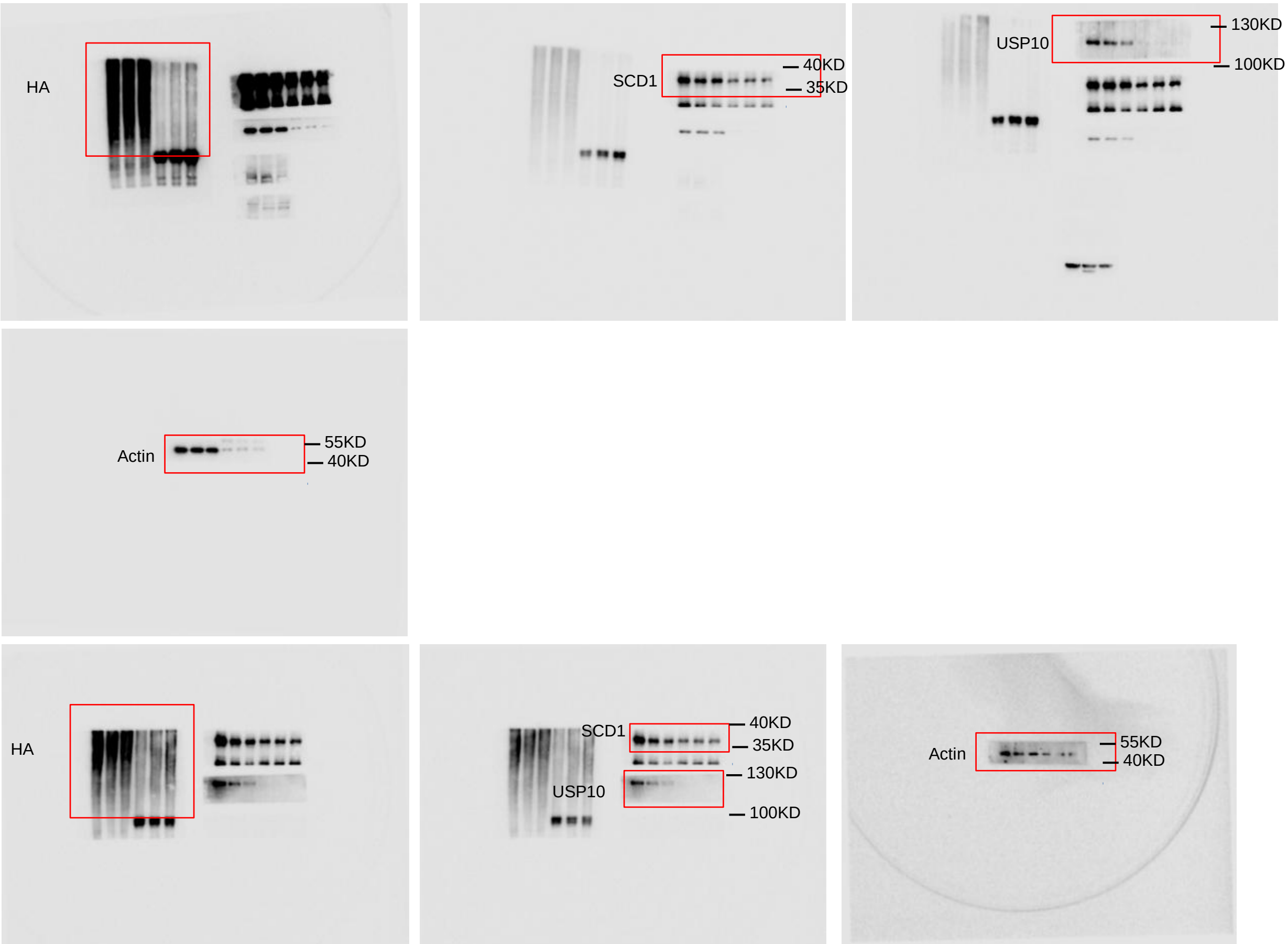

Figure 7J

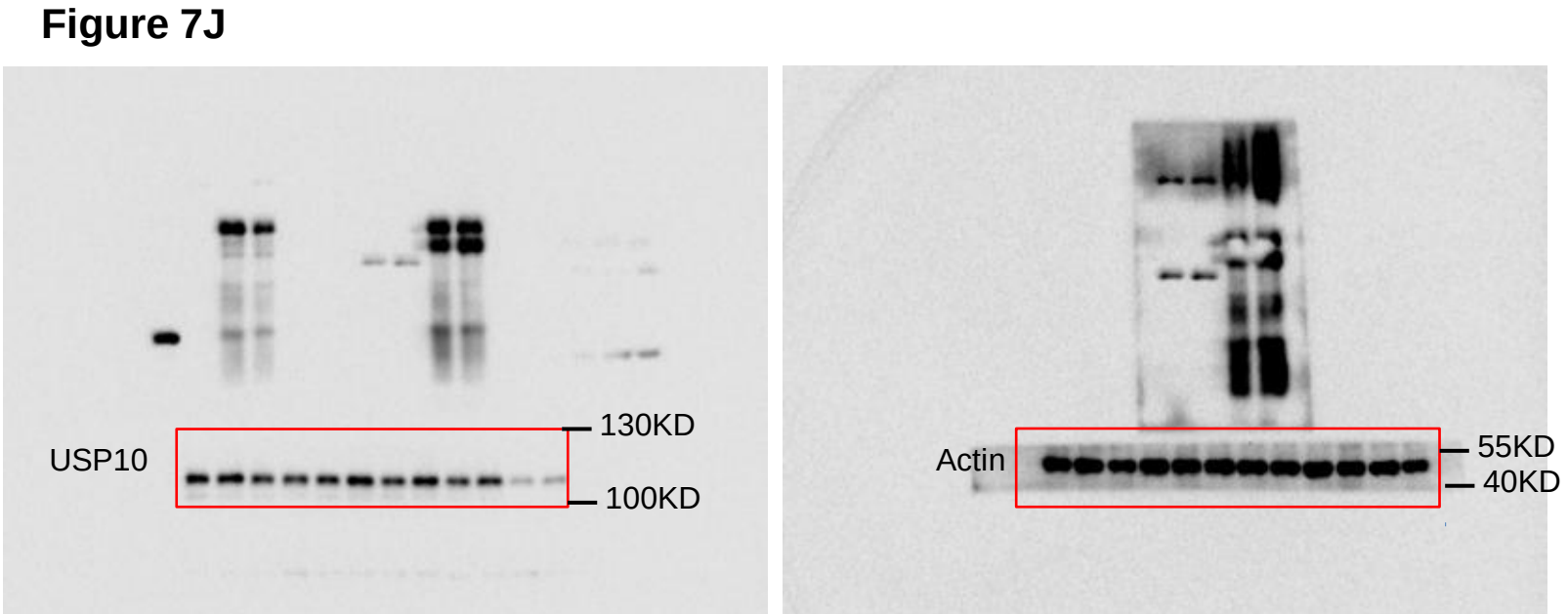

UM-SCC1

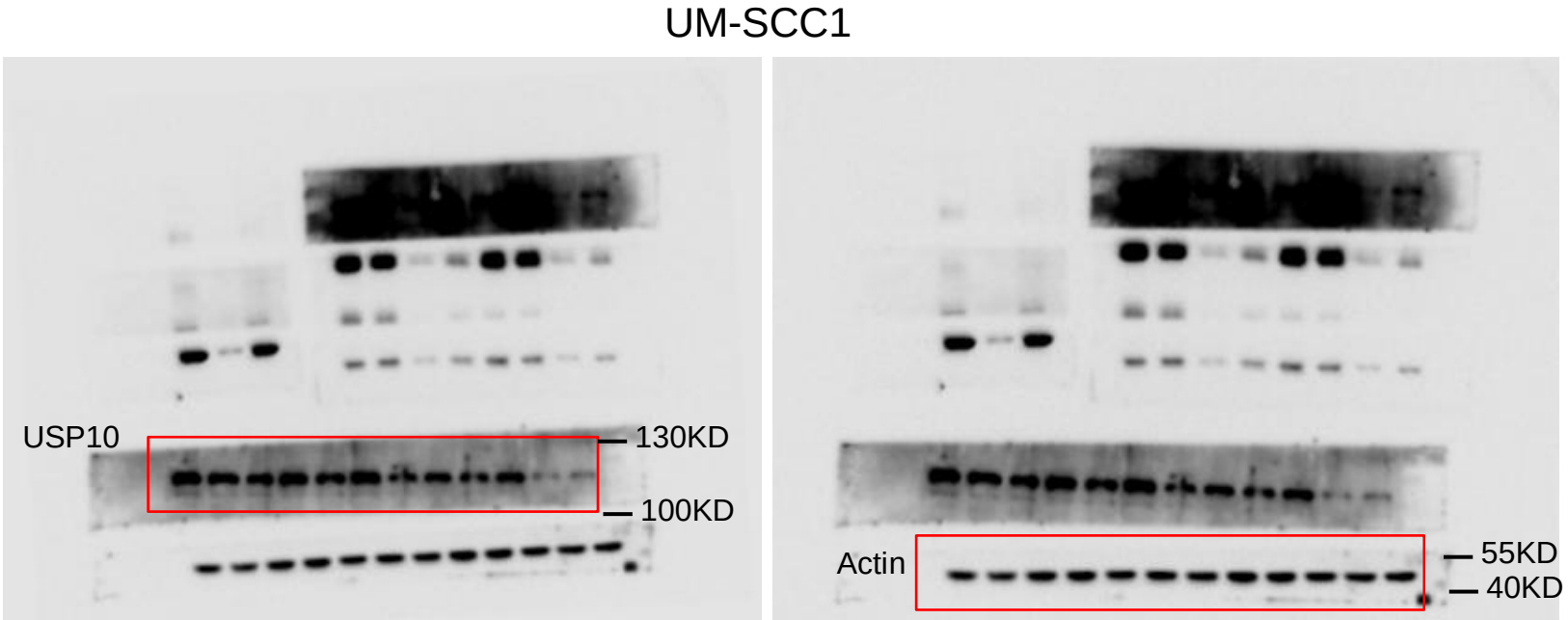

UM-SCC6

Figure 9

Figure 9F

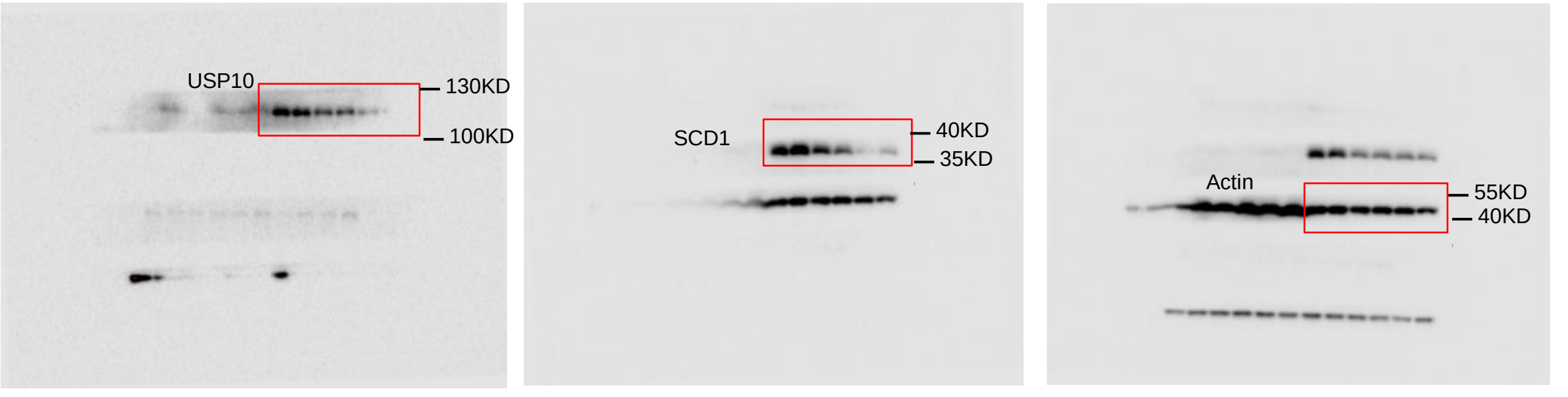

Supplementary Figure 1

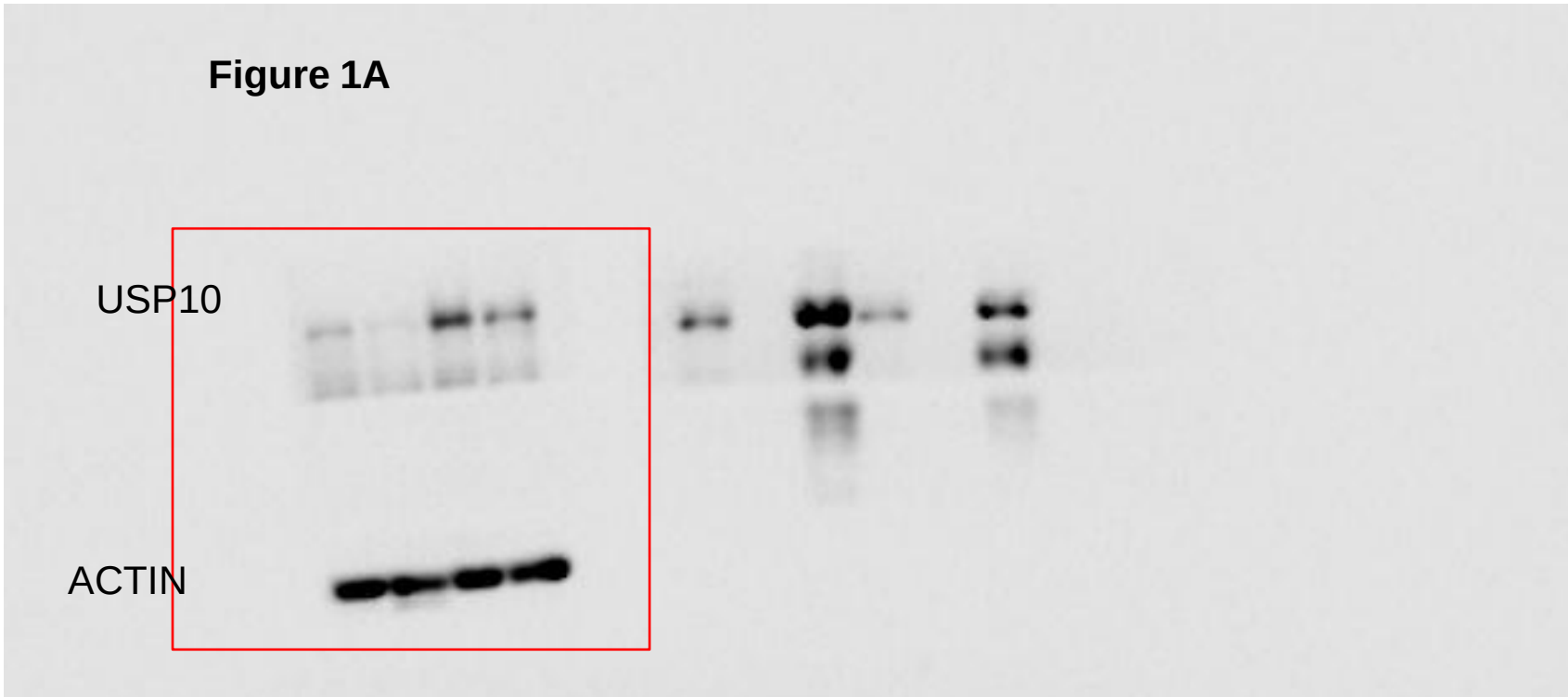

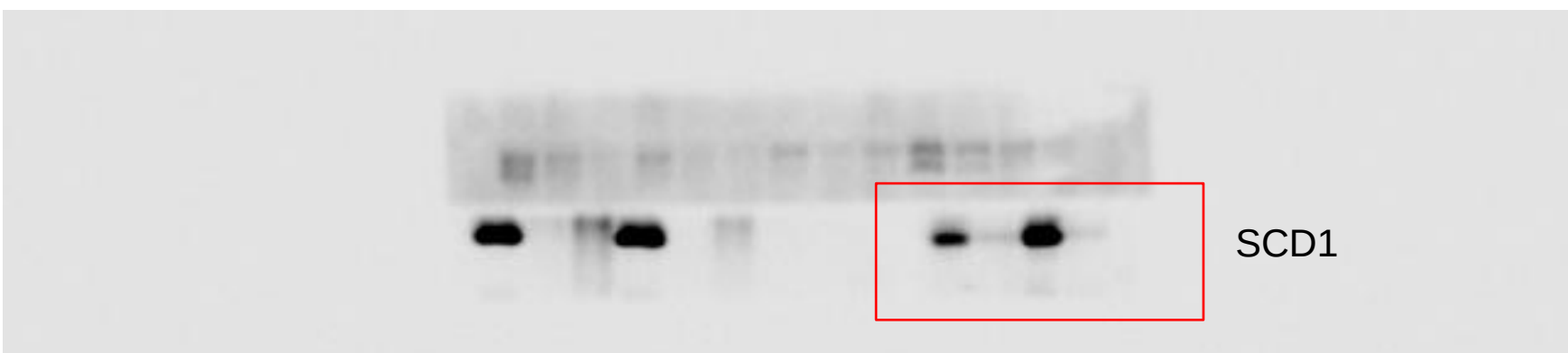

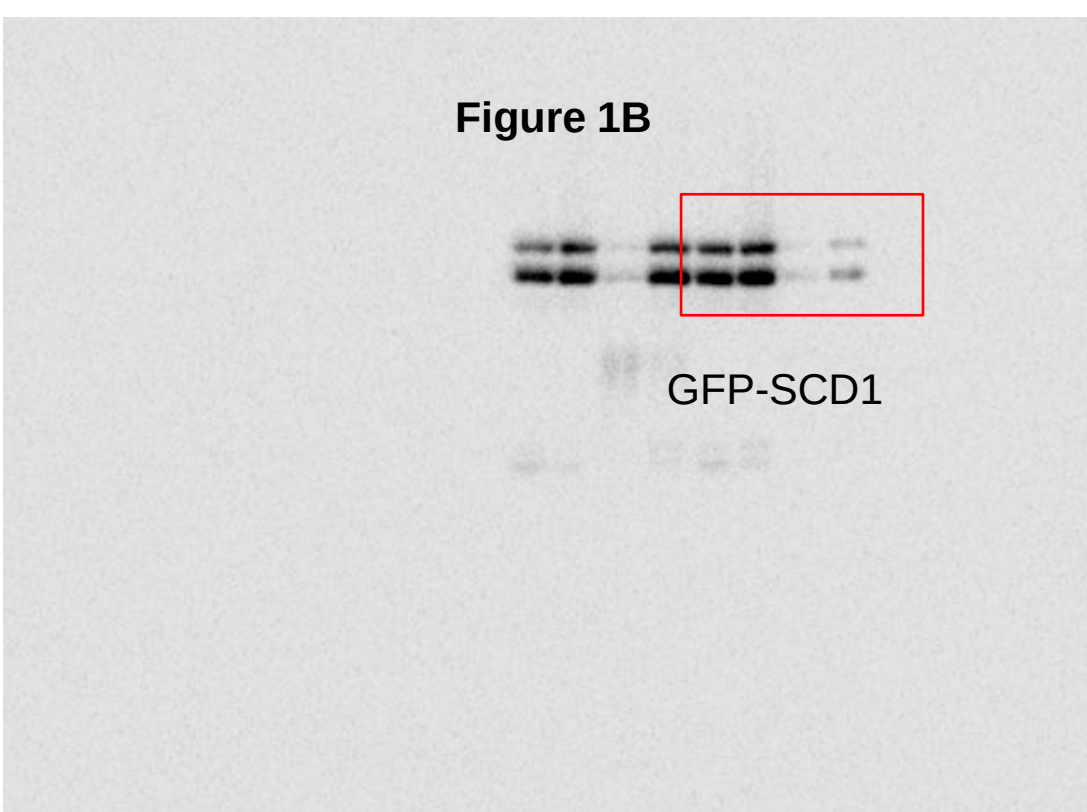

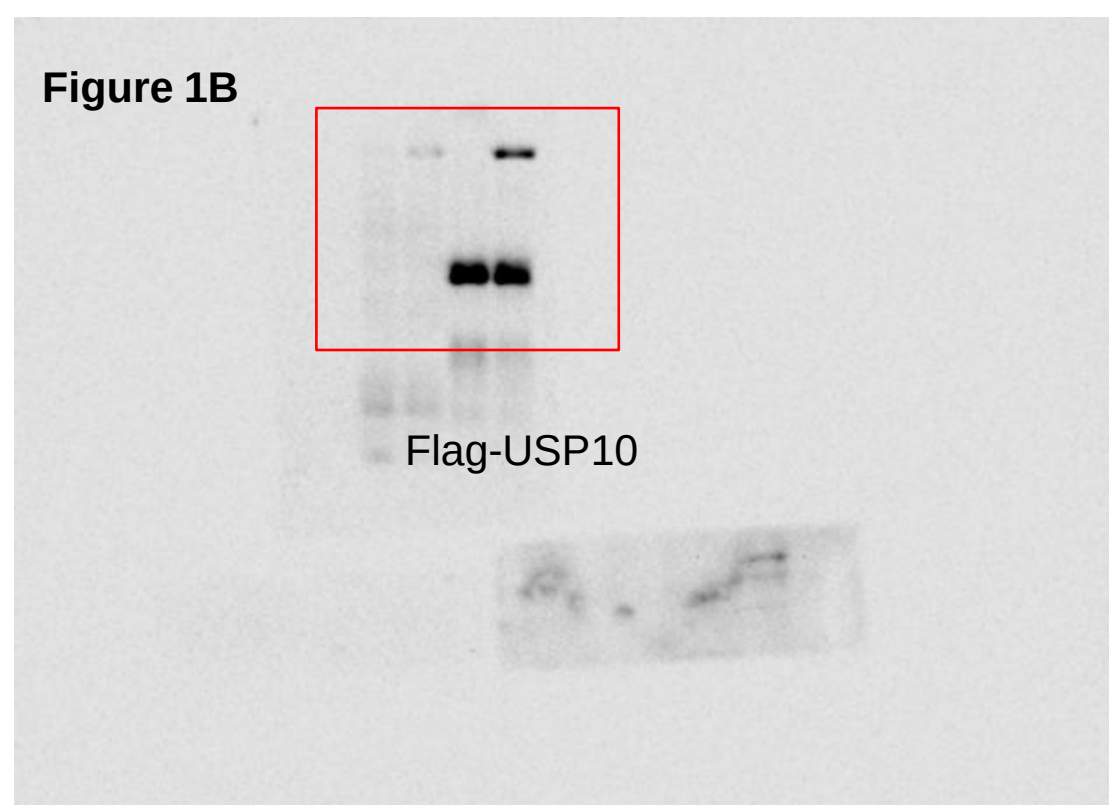

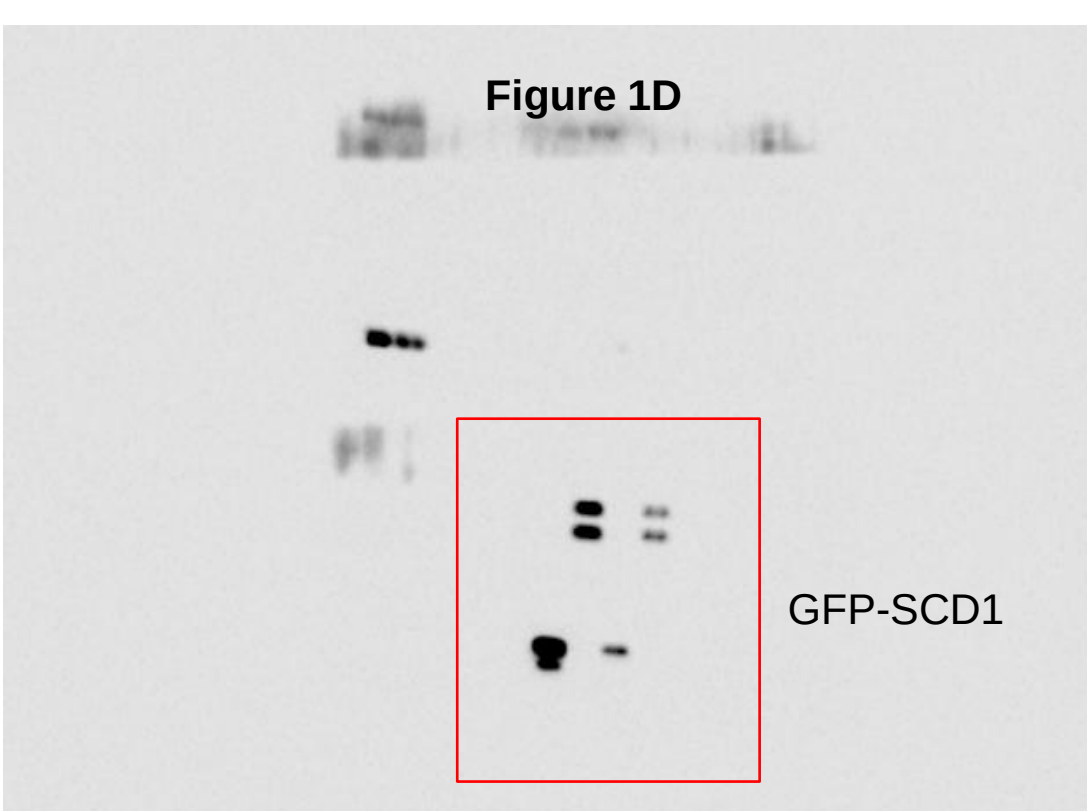

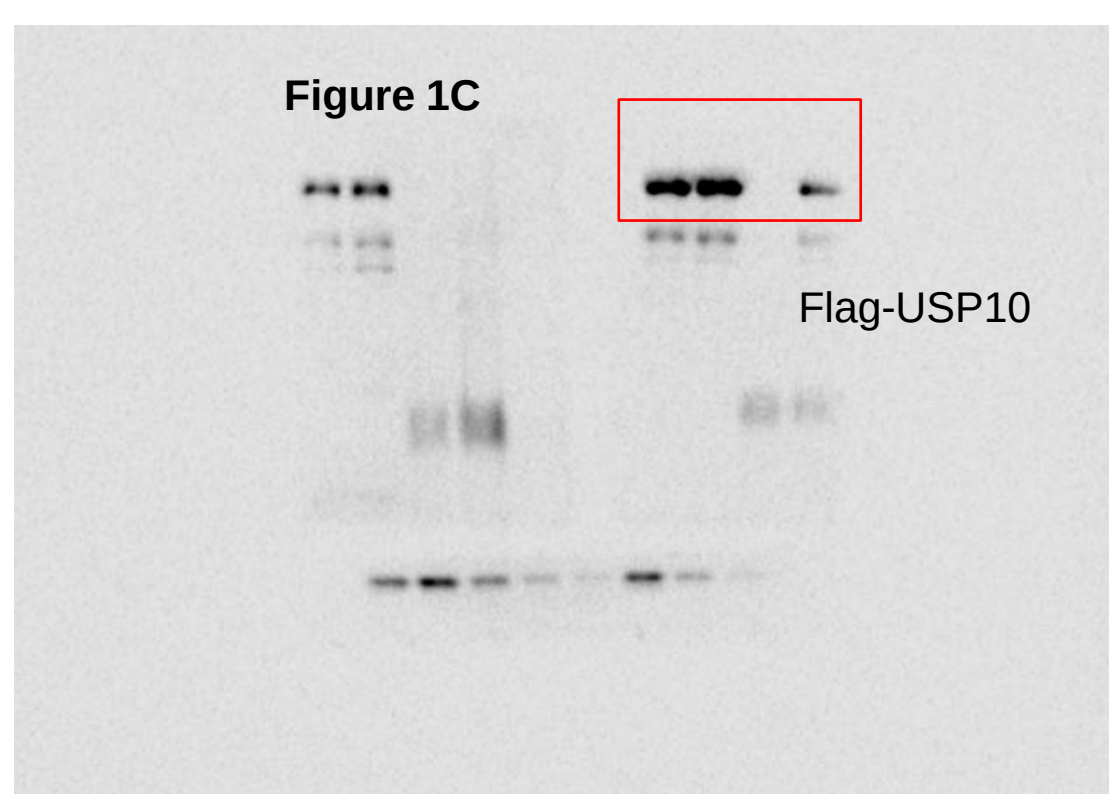

Supplementary Figure 2

Supplementary Figure2B

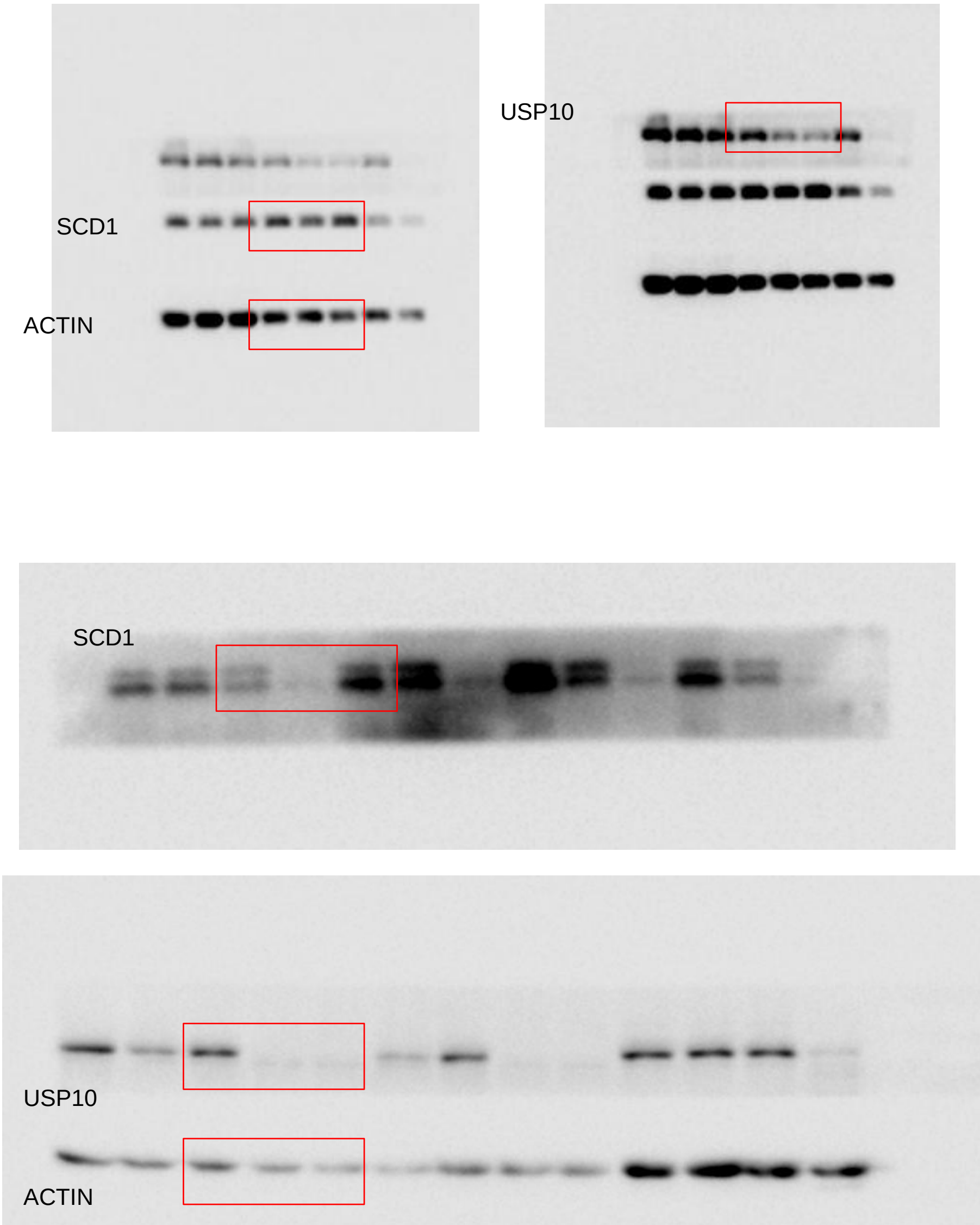

Supplementary Figure3A

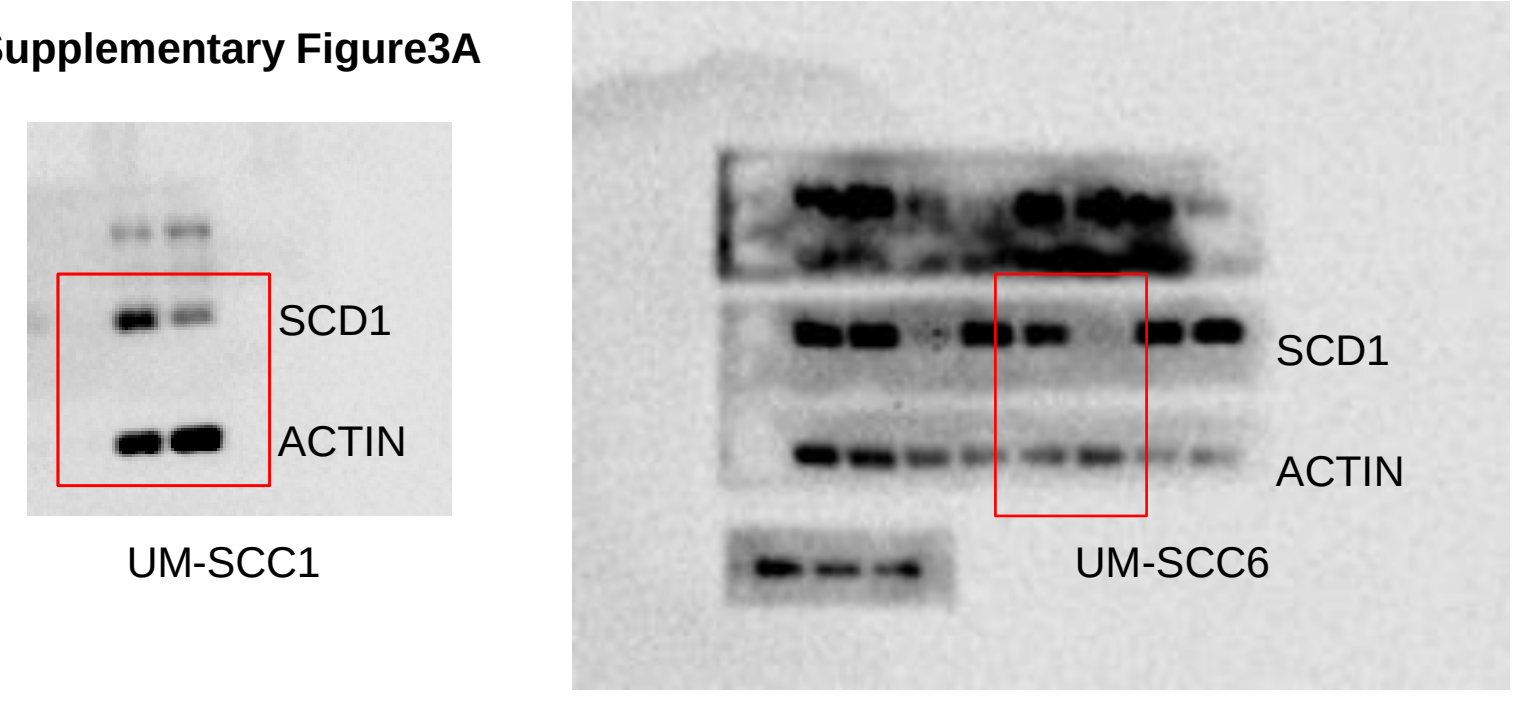

Supplementary Figure 4

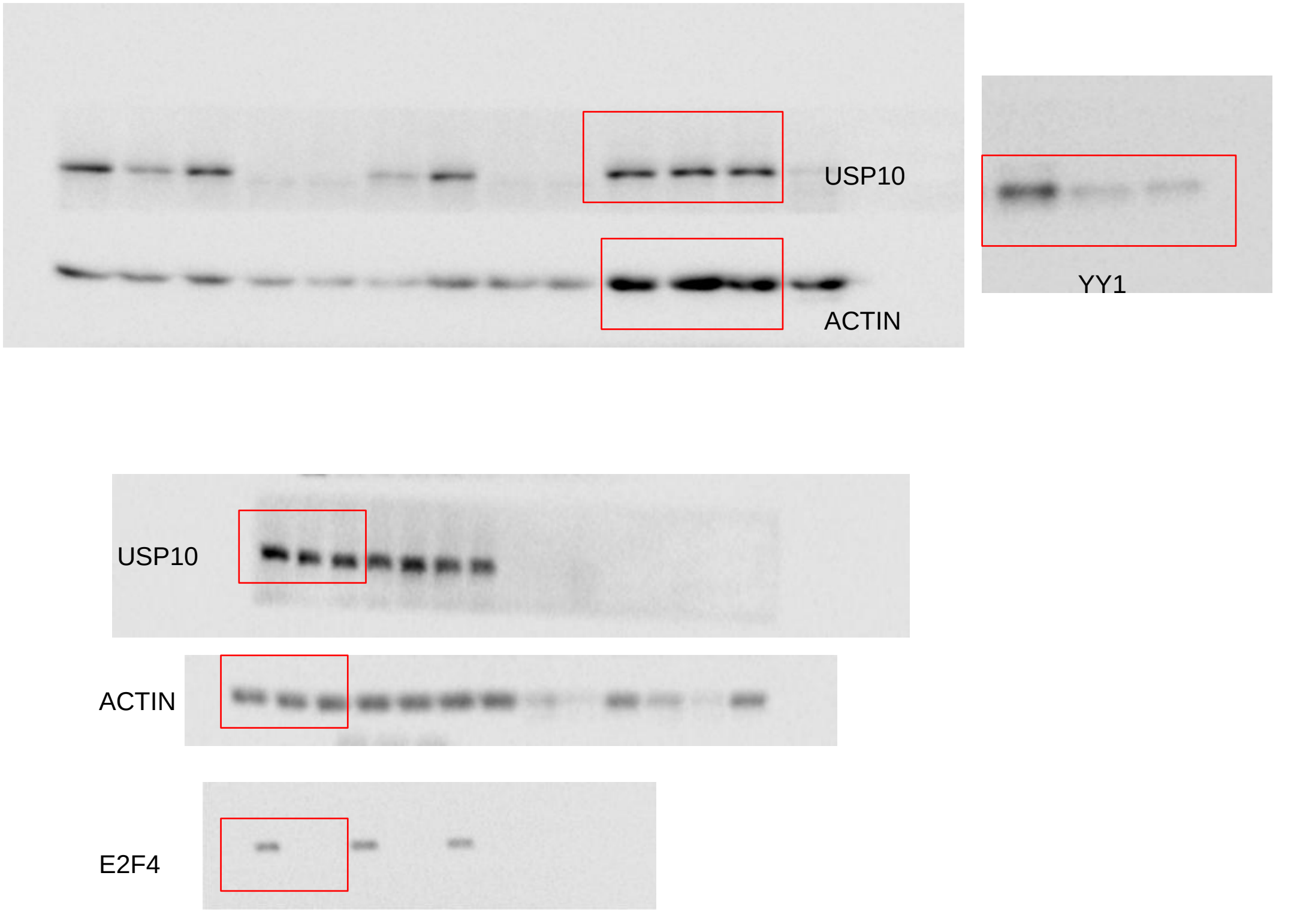

Supplementary Figure 5

Supplementary Figure5A

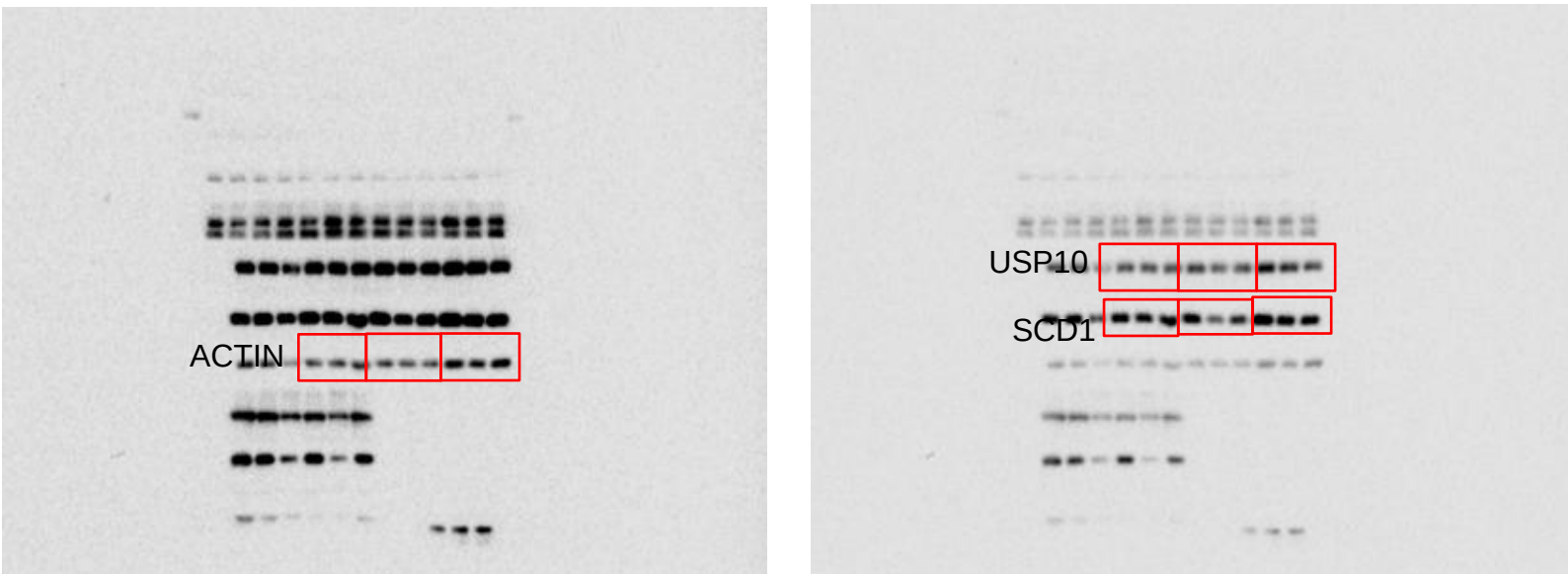

Golvatinib Altiratinib Zoliflodacin

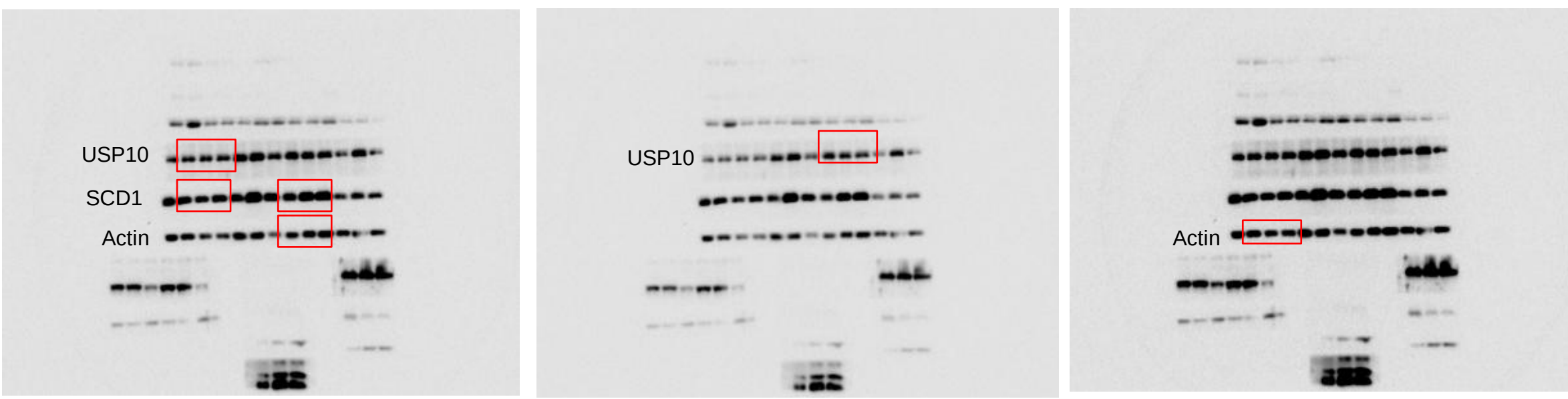

Delamanid PCO-371

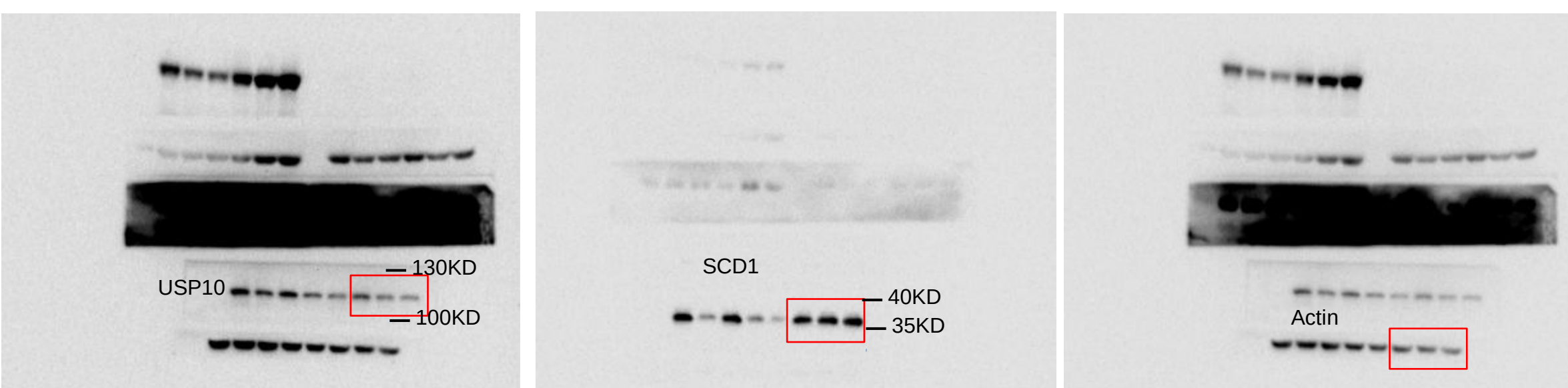

Bemcentinib

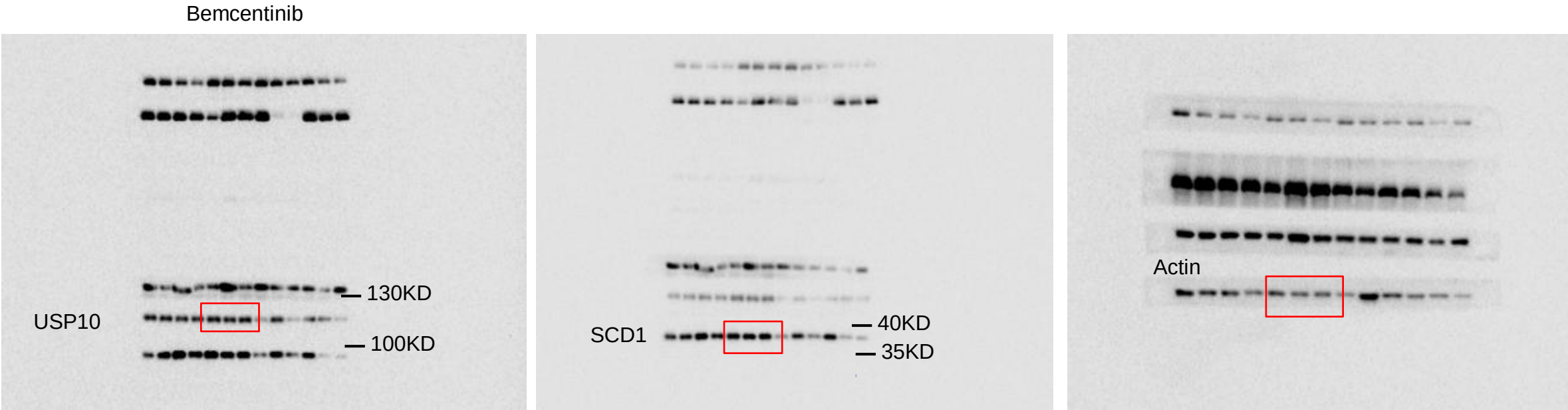

MK-3207

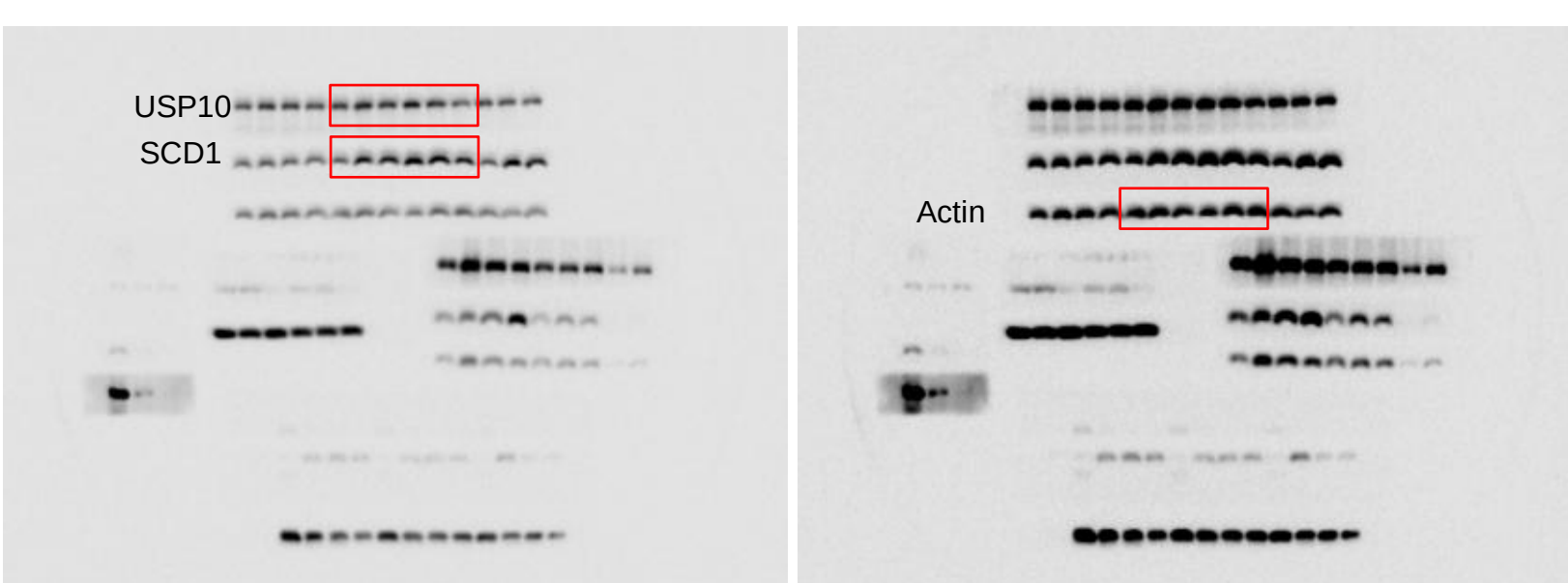

Entrectinib AZD8529
