# Supplementary material for: Targeting USP10/SCD1 axis by RAF265 suppresses lipogenesis and induced ferroptosis in head and neck squamous cell carcinoma
Source: Cell Death Discov. 2026 Jun 2;12:325. doi: 10.1038/s41420-026-03180-1 (PMC13444109; doi:10.1038/s41420-026-03180-1)
Supplement: Supplementary file 2 [file 41420_2026_3180_MOESM1_ESM.docx]

**Supplementary Figures and Figure legends**

**Supplementary Fig.1** (A) CAL-27 and UM-SCC1 cells were treated with Spautin-1for 36h. The expression of SCD1 and USP10 were detected by Western blot. (B–C) Co-transfection of Flag-tagged USP10 and GFP-tagged SCD1 into 293T cells was conducted, followed by immunoprecipitation using anti-Flag or anti-GFP antibodies to examine their interaction.

**Supplementary Fig.2** (A) USP10-knockdown and control UM-SCC1 cells were treated with RSL3 alone or in combination with Ferrostatin-1 (Ferr-1), Z-VAD-FMK, or 3-MA, followed by assessment of cell viability.(B) SCD1 was overexpressed in HNSCC cells with or without USP10 knockdown. The expression levels of USP10 and SCD1 were detected by Western blot. Data in panel A was representative results of three independent experiments and analyzed using Student’s t-test; *p < 0.05, **p < 0.01, ***p < 0.001.

**Supplementary Fig.3** (A) SCD1 was knocked down in UM-SCC1 and UM-SCC6 cells. The protein levels of SCD1 were detected by Western blot. (B-E) Cell proliferation and migration were assessed by colony formation and Transwell assays. (F) IHC staining of tumor sections was performed using an anti-4NHE antibody. (G-H) Immunohistochemical staining of normal tissues(n=9) and HNSCC tissues(n=45) using SCD1 antibody, followed by evaluation of SCD1 expression. Data in panels C and E were representative results of three independent experiments and analyzed using Student’s t-test; *p < 0.05, **p < 0.01, ***p < 0.001.

**Supplementary Fig.4** **(A)** The 25 topped correlated genes with USP10 in HNSCC were obtained from Ualcan database. (B-C) The correlation between YY1 and USP10 in HNSCC was analyzed using Ualcan and GEPIA database. (D-E) Western blot and qRT-PCR were used to investigate the expression levels of USP10 in YY1 depleting UM-SCC1 cell. (F) Western blot was used to investigate the expression levels of USP10 in E2F4 depleting CAL-27 cell.

**Supplementary Fig.5** (A) UM-SCC1 cells were treated with the indicated inhibitors and the expression levels of USP10 and SCD1 were analyzed. (B) Western blot analysis was used to examine protein levels of SCD1 and USP10 in Cal-27 cells treated with 0, 2, or 3 µM RAF265 for 24 hours. (C) The levels of oleic acid and palmitoleic acid were determined by lipidomic mass spectrometry inUM-SCC1 cells with or without RAF265 treatment . Data in panel C was representative results of three independent experiments and analyzed using Student’s t-test; *p < 0.05, **p < 0.01, ***p < 0.001.

**Supplementary Fig.6** (A-C) Cal-27 cells were treated with 0,2 and 3μM RAF265. Cell growth and migration were analyzed using colony formation and transwell assays.

Data in panels C and D were representative results of three independent experiments and analyzed using Student’s t-test; *p < 0.05, **p < 0.01, ***p < 0.001.
